# Atypical behavioral and thermoregulatory circadian rhythms in mice lacking a microbiome

**DOI:** 10.1038/s41598-022-18291-9

**Published:** 2022-08-25

**Authors:** Vanessa A. Leone, Kenneth G. Onishi, Megan Kennedy, Jonathan P. Riggle, Joseph F. Pierre, Andrew C. Maneval, Melanie N. Spedale, Betty R. Theriault, Eugene B. Chang, Brian J. Prendergast

**Affiliations:** 1 Department of Animal and Dairy Sciences, University of Wisconsin-Madison, 1933 Observatory Dr., Madison, WI 53706 USA; 2 Department of Medicine, University of Chicago, Chicago, IL 60637 USA; 3 Department of Psychology, Institute for Mind and Biology, University of Chicago, 940 E 57th St., Chicago, IL 60637 USA; 4 Medical Scientist Training Program, University of Chicago, Chicago, IL 60637 USA; 5 Department of Ecology and Evolution, University of Chicago, Chicago, IL 60637 USA; 6 Department of Nutritional Sciences, University of Wisconsin-Madison, Madison, WI 53706 USA; 7 Animal Resources Center, The University of Chicago, Chicago, IL 60637 USA; 8 Department of Surgery, University of Chicago, Chicago, IL 60637 USA

**Keywords:** Microbiology, Neuroscience, Endocrinology

## Abstract

Trillions of microbial oscillators reside throughout the mammalian body, yet their contributions toward fundamental features of host circadian rhythms (CRs) have not been characterized. Here, we demonstrate that the microbiome contributes to host CRs in activity and thermoregulation. Mice devoid of microbes (germ-free, GF) exhibited higher-amplitude CRs in a light–dark cycle and longer circadian periods in constant darkness. Circadian entrainment to food was greater in GF mice, but resetting responses to simulated jet-lag were unaffected. Microbial transplantation with cecal contents of conventionally-raised mice normalized CRs of GF mice, indicating that the concurrent activity of gut microbes modulates host circadian networks. Obesogenic effects of high-fat diet were absent in GF mice, but some circadian-disruptive effects persisted. Transkingdom (host-microbe) interactions affect circadian period and entrainment of CRs in diverse traits, and microbes alter interactions among light- and food-entrainable circadian processes in the face of environmental (light, diet) perturbations.

## Introduction

Mammalian circadian rhythms are generated by a hierarchically-organized network of cell- and tissue-level circadian oscillators^1^. A pacemaker in the hypothalamus occupies a superordinate position in this hierarchy, generating a circadian period, entraining to environmental light–dark cycles, and setting the period and phase of rhythmic and non-rhythmic tissues in the periphery^1^. Peripheral circadian oscillators do not entrain directly to light, but instead rely on time cues from the pacemaker and from pacemaker-driven processes (e.g., behavior, hormone secretion) in order to maintain appropriate intra-cellular and intra-tissue phase^2^. Collectively, the circadian pacemaker and peripheral oscillators comprise an organismal circadian network^3–5^, which maintains phase with the external environment and coordinates rhythm coherence within the internal milieu^2,6^.

Upon and within the mammalian body reside trillions of microbes^7^. Diurnal rhythms in host metabolism and in the timing of food intake interact to drive daily microbiome rhythms^8–10^. Microbiomes are shaped by, but may also contribute to, daily circadian dynamics of the host through temporal mutualism^11,12^. For example, algal symbionts modify daily behavioral rhythms in sea anemone^13^, and bioluminescent bacteria confer circadian function upon the light organ of bobtail squid^14^. Mounting evidence indicates mammalian gut microbes play prominent roles in host homeostatic processes (e.g., digestion, metabolism, and immune function^15,16^). Whereas disruptions to the host circadian system markedly dysregulate the microbiome^10^, the extent to which microbes participate in expression of organismal-level circadian rhythms in mammals has not been established, and functional properties of the circadian system absent a microbiome have not been explicitly examined. Thus, we tested the hypothesis that the microbiome is a necessary component of the mammalian host circadian network.

## Results

### The microbiome contributes to entrainment and generation of circadian rhythms

**Figure 1:**
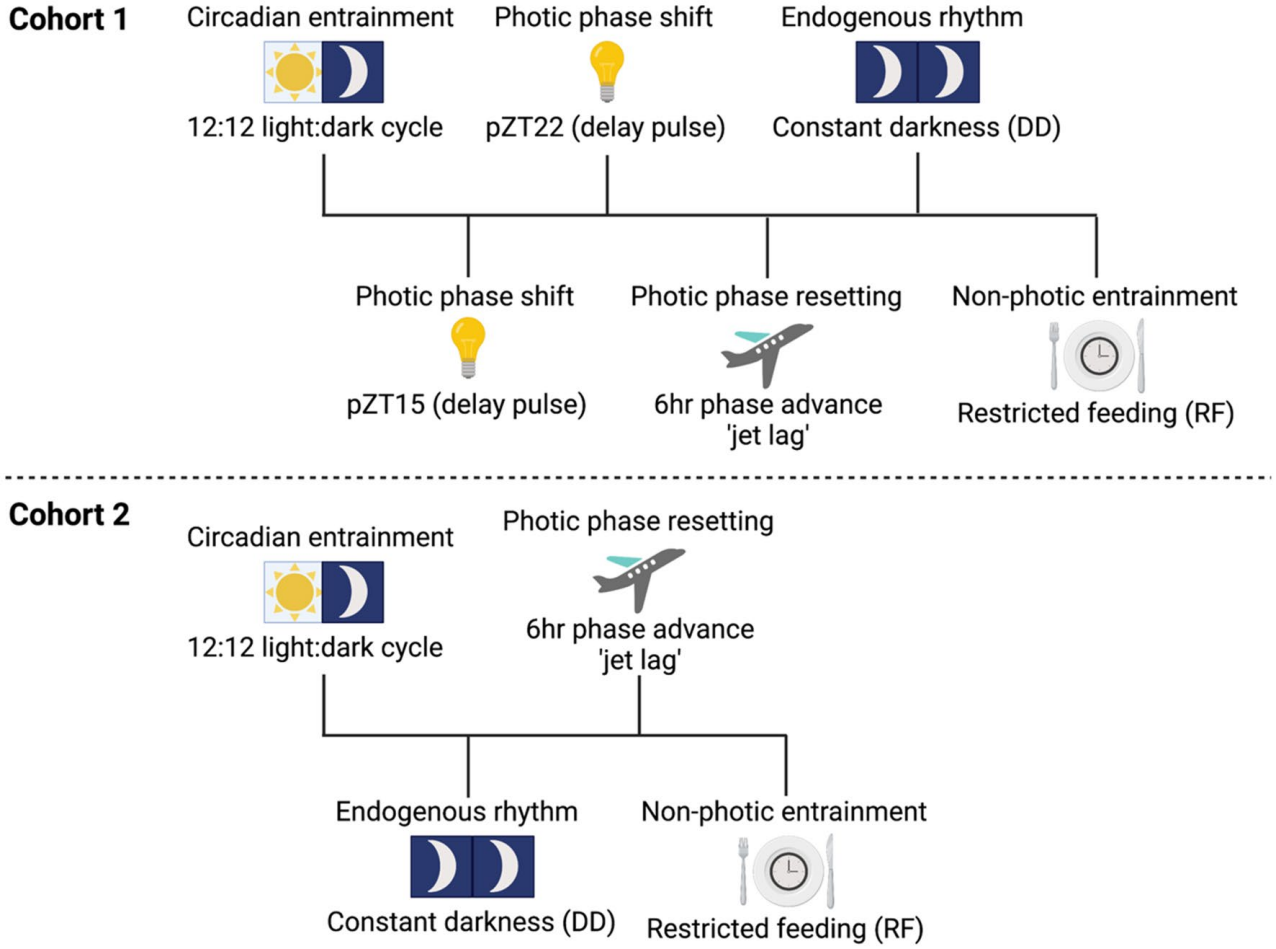
Circadian manipulations and behavioral data collection. Data collection were conducted in two experimental cohorts. In Cohort 1, the order of circadian manipulations was as follows: Circadian entrainment to 12L:12D, Photic phase resetting (jet lag), Photic phase shift [acute light pulses at projected ZT15 (‘pZT15’), then at pZT22], Endogenous rhythm in constant darkness (DD), and Non-photic food entrainment (restricted feeding; RF). In Cohort 2, the order of circadian manipulations was as follows: Circadian entrainment to 12L:12D, Endogenous rhythm in DD, Photic phase resetting (jet lag), and Non-photic food entrainment (RF). Data sets from replication experiments were combined only if no statistically significant differences were observed between cohorts, which occurred in all instances except in the case of the photic phase resetting (jet lag) test (F_1,319_ = 69.318, P < 0.0001), therefore these data were reported separately for each cohort (see Fig. S5). Created with BioRender.com.

We first documented how the presence of gut microbes impacted two temporal features fundamental to all circadian systems: (1) entrainment, or the ability to synchronize mammalian organismal circadian rhythms to their primary *Zeitgeber*, the environmental light–dark cycle; and (2) self-sustained rhythmicity, or the capacity to generate and maintain circadian rhythms in multiple traits absent environmental input. To examine the necessity of the microbiome in these regards, mice devoid of all microbes [‘germ-free’ (GF) mice] were housed in flexible film isolators to maintain sterility while also permitting radio telemetry monitoring of home-cage locomotor activity (LMA) and core body temperature (T_b_) via surgically-implanted i.p. transmitters. Recordings were also obtained from conventionally-raised control mice [‘specific pathogen free’ (SPF) mice] housed in the same rooms but outside the isolators. This allowed use of lighting and feeding manipulations to elicit changes in circadian behavior (Fig. 1) while maintaining all groups under identical conditions of light, temperature, and humidity.

**Figure 2:**
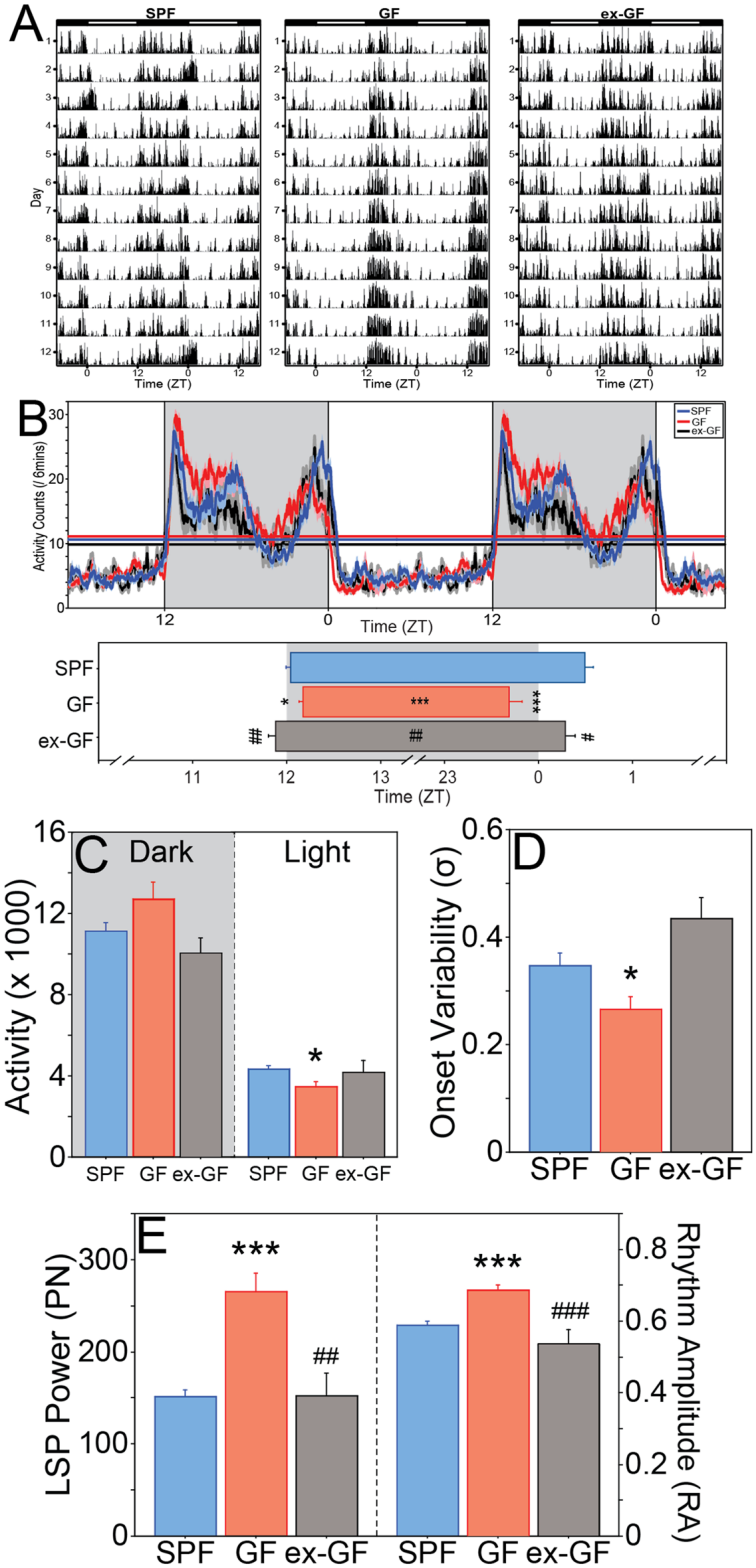
Entrainment of the circadian locomotor activity rhythm to light is altered in the absence of a microbiome. (**A**) Double-plotted home-cage locomotor activity (LMA) record of representative SPF (left panel), GF (middle panel) and ex-GF (right panel) mice housed in a 12L:12D photocycle (LD). Time is indicated on the horizontal axis, as are light (white) and dark (black) phases of the LD photocycle. (**B**) Double-plotted LMA waveform profile (mean ± SEM daily activity in 6 min bins) of SPF, GF, and ex-GF mice in LD (top panel); mean + SEM onset and offset of daily locomotor activity of SPF, GF and ex-GF mice in LD (bottom panel); onset and offset are represented by the beginning and end of each bar. (**C**) Mean + SEM total activity counts of SPF, GF and ex-GF mice in the dark (shaded) and light (unshaded) phases of the LD cycle. (**D**) Mean + SEM variability in activity onsets of SPF, GF and ex-GF mice in LD. (**E**) Mean + SEM circadian power (left panel) and rhythmic amplitude (right panel) of SPF, GF and ex-GF mice in LD. SPF: n = 18; GF: n = 16; ex-GF: n = 6. *P < 0.05, ***P < 0.001 v SPF mice; ^#^P < 0.05, ^##^P < 0.01, ^###^P < 0.001 v GF mice.

**Figure 3:**
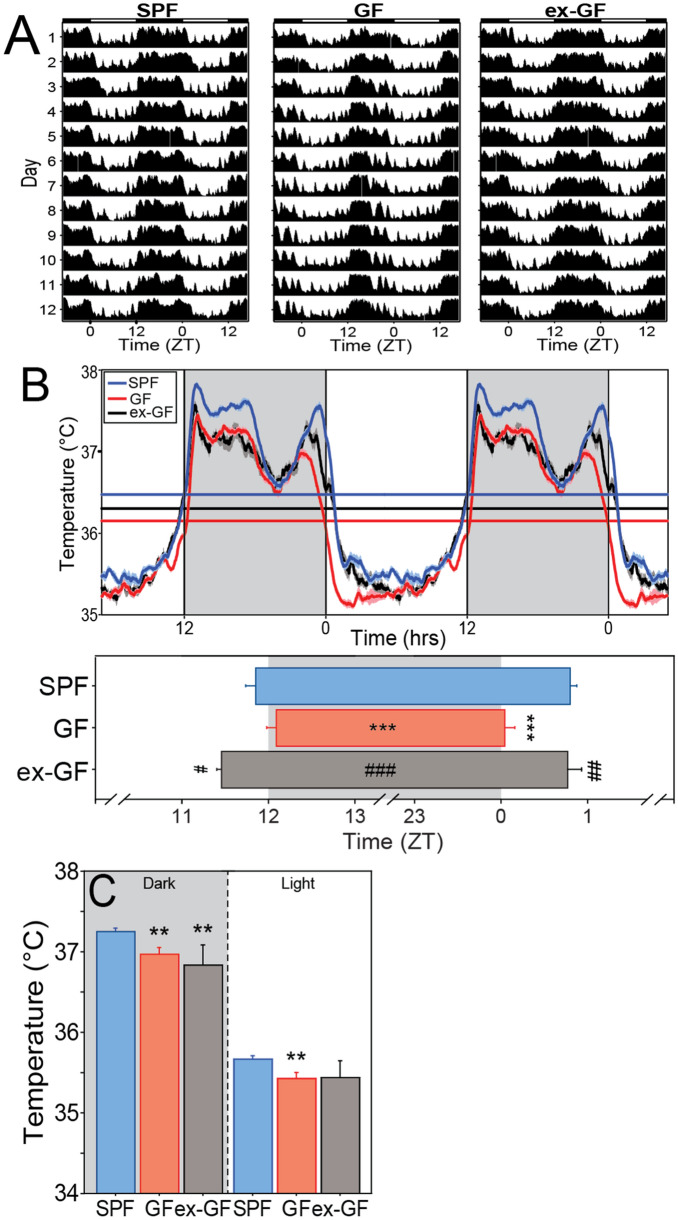
Circadian rhythms and homeostatic maintenance of core body temperature depend on the microbiome. (**A**) Representative, double-plotted records of core body temperature (T_b_) in SPF (left panel), GF (middle panel), and ex-GF (right panel) mice housed in a 12L:12D photocycle (LD). Actogram annotations as in Fig. 2. (**B**) Double-plotted T_b_ waveform profile (mean ± SEM T_b_ at 6 min intervals) of SPF, GF, and ex-GF mice in LD (top panel); mean + SEM beginning and end of nightly elevated T_b_ in SPF, GF and ex-GF mice in LD (bottom panel). (**C**) Mean + SEM T_b_ of SPF, GF and ex-GF mice in the dark (shaded) and light (unshaded) phases of the LD cycle. SPF: n = 18; GF: n = 15; ex-GF: n = 4. **P < 0.01, ***< 0.001 v SPF mice; ^#^P < 0.05, ^##^P < 0.01, ^###^P < 0.001 v GF mice.

GF mice exhibited atypical LMA and T_b_ in a 12L:12D photocycle (LD; Figs. 2, 3 and S1). A component of circadian LMA normally associated with the end of the scotophase (dawn) occurred earlier and was substantially decreased in GF mice (e.g., Fig. 2A middle panel, B). Nocturnal activity began ~ 10 min later (t_32_ = 2.055, P = 0.0481) and ended ~ 1 h earlier in GF compared to SPF mice, (t_32_ = 4.984, P < 0.0001; Fig. 2B), resulting in a markedly compressed interval of nocturnal activity (α_LMA_; 12.5 ± 0.09 h vs. 11.5 ± 0.1 h; t_32_ = 5.920, P < 0.0001; Fig. 2B). Total daily activity levels were comparable between SPF and GF mice (t_32_ = 0.618, P = 0.5409), but daytime-specific activity was 20% lower in GF mice (t_32_ = 2.836, P = 0.0079; Fig. 2C). The timing of activity onset each day was significantly less variable in GF mice (t_32_ = 2.439, P = 0.0205; Fig. 2D). Overall, circadian rhythm power and amplitude were substantially greater in GF mice (power: t_32_ = 5.566, P < 0.0001; amplitude: t_32_ = 5.472, P < 0.0001; Fig. 2E). A similar circadian waveform was evident in T_b_ (Figs. 3A,B and S2): the morning decline in T_b_ occurred significantly earlier in GF mice (t_31_ = 5.539, P < 0.0001; Fig. 3B), such that the net interval of elevated T_b_ (α_Tb_) was ~ 1 h shorter (t_31_ = 5.479, P < 0.0001; Fig. 3B). GF mice were also hypothermic: T_b_ was ~ 0.25 °C lower in GF mice throughout the diurnal cycle (α_Tb_ t_31_ = 2.983, P = 0.0055; ρ_Tb_ t_31_ = 2.814, P = 0.0084; α_Tb_ & ρ_Tb_ collapsed t_31_ = 3.869, P = 0.0005; Fig. 3C).

**Figure 4:**
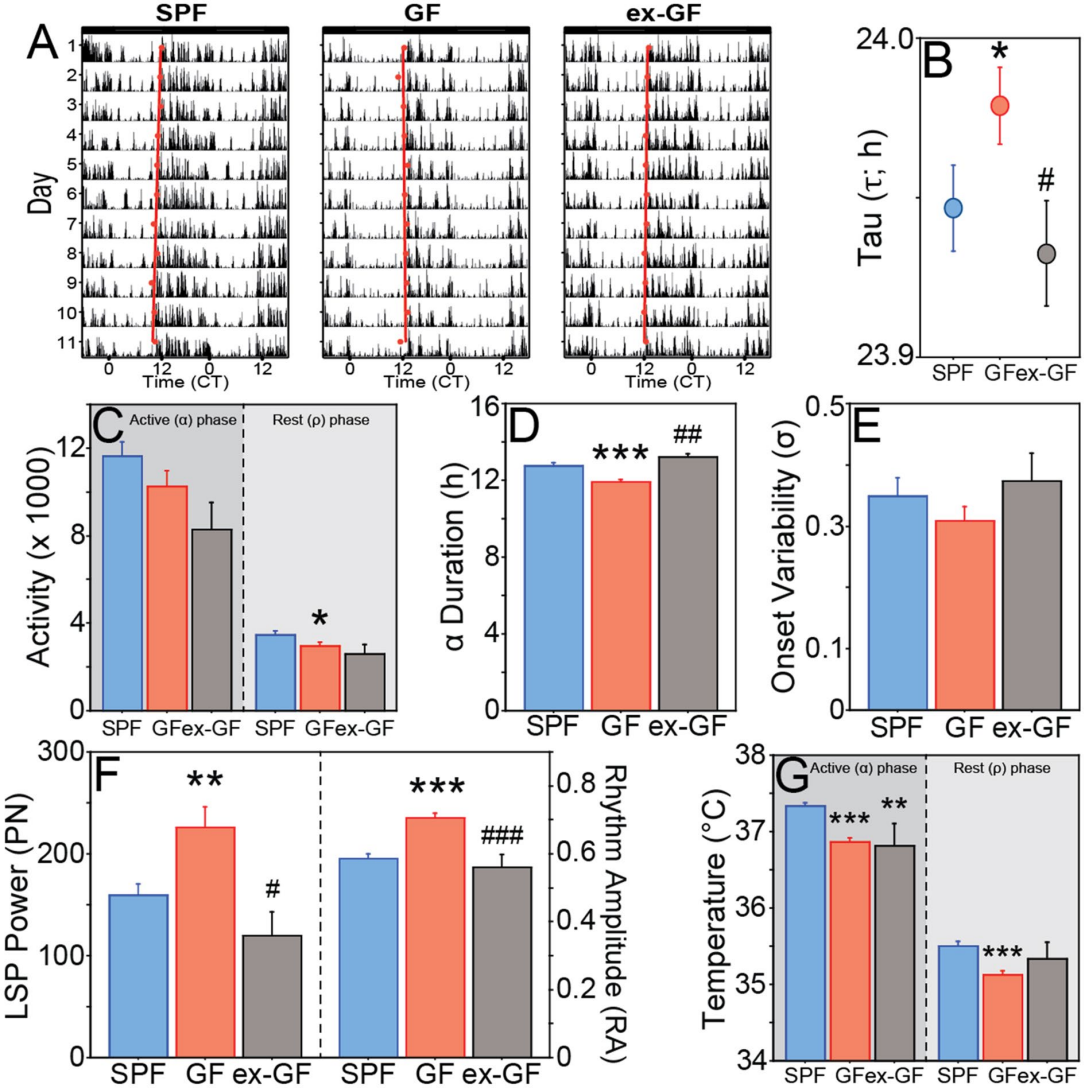
Expression of free-running circadian rhythms are altered in the absence of microbes. (**A**) Representative, double-plotted home-cage locomotor activity (LMA) record of SPF (left panel), GF (middle panel) and ex-GF (right panel) mice housed in continuous darkness (DD). Activity onsets are depicted with red circles, over which a regression line is plotted for the calculation of free-running circadian period (τ). (**B**) Mean ± SEM period (in h) of the free running circadian rhythm of LMA in DD. (**C**) Mean + SEM total activity counts during the subjective night (active phase; dark shading) and subjective day (inactive phase; lighter shading) of SPF, GF and ex-GF mice in DD. (**D**) Mean + SEM duration of the active phase during each circadian cycle (α_LMA_) of SPF, GF and ex-GF mice in DD. (**E**) Mean + SEM variability in activity onsets of SPF, GF and ex-GF mice in DD. (**F**) Mean + SEM circadian power (left panel) and rhythmic amplitude (right panel) of SPF, GF and ex-GF mice in DD. (**G**) Mean + SEM T_b_ of SPF, GF and ex-GF mice in DD during the subjective night (active phase; dark shading) and subjective day (inactive phase; lighter shading). LMA SPF: n = 18; GF: n = 16; ex-GF: n = 5. T_b_ SPF: n = 18; GF: n = 15; ex-GF: n = 4. *P < 0.05, **P < 0.01, ***P < 0.001 v SPF mice; ^#^P < 0.05, ^###^P < 0.001 v GF mice.

Following transfer into constant darkness (DD), all mice expressed free-running LMA and T_b_ rhythms (Fig. 4A and Fig. S3). The period (τ) of the circadian LMA rhythm was significantly longer in GF compared to SPF mice (t_32_ = 2.055, P = 0.0481; Fig. 4B), and although the magnitude of this difference in τ was only 2–3 min/day, the effect size was medium-large (Hedges’ *g* = 0.7). Total activity counts remained comparable in GF and SPF mice (t_32_ = 1.580, P = 0.1240), but GF mice were less active during the rest (ρ) phase (i.e., subjective daytime, t_32_ = 2.294, P = 0.0285; Fig. 4C), and the duration of the active phase (α_LMA_) was > 1 h shorter in GF mice (t_32_ = 5.397, P < 0.0001; Fig. 4D). In the absence of a LD cycle (i.e., in DD), GF mice no longer exhibited greater day-to-day precision in the timing of activity onset (t_32_ = 1.026, P = 0.3126; Fig. 4E), but circadian rhythm power and amplitude were greater in GF relative to SPF mice (power: t_32_ = 2.867, P = 0.0073; amplitude: t_32_ = 5.656, P < 0.0001; Fig. 4F). Period values for T_b_ were strikingly similar to those for LMA, but the effect of microbes was not statistically significant (t_31_ = 1.806, P = 0.0806; not illustrated). As in LD, α_Tb_ was shorter in GF mice in DD (11.9 ± 0.1 h vs. 12.7 ± 0.2 h; t_31_ = 3.554, P = 0.0012; not illustrated); GF mice were also globally hypothermic in DD (t_31_ = 7.677, P < 0.0001); hypothermia was not merely due to the shorter interval of elevated nightly T_b_: T_b_ was lower in GF mice during both phases of the circadian cycle (subjective day, t_31_ = 4.749, P < 0.0001; subjective night, t_31_ = 6.430, P < 0.0001; Fig. 4G).

**Figure 5:**
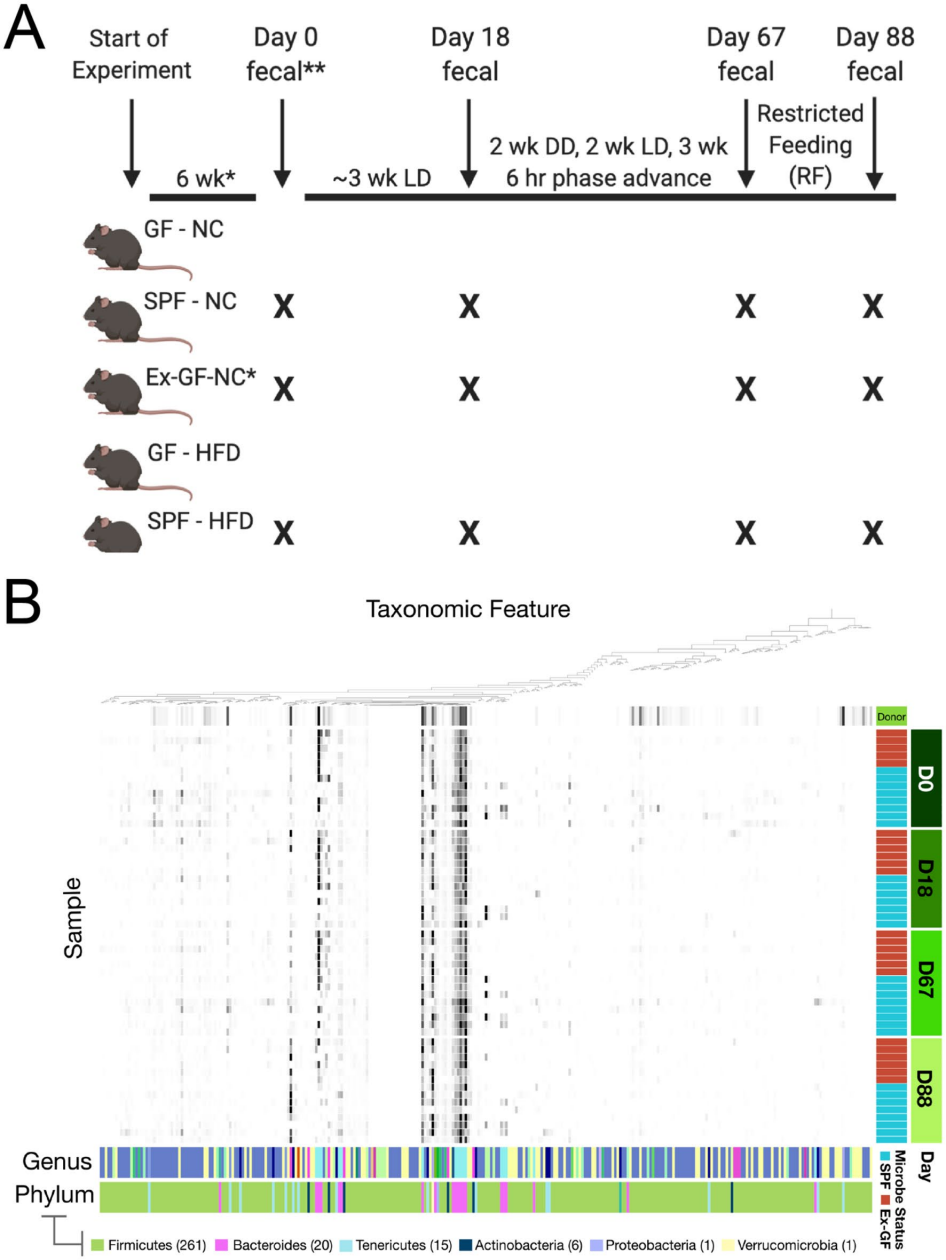
Fecal collection for 16S rRNA gene sequencing analysis and study timeline. **(A)** Fecal collection timeline. Asterisk: Six weeks prior to day 0 (D0), ex-GF mice were conventionalized. After 4 weeks, GF, ex-GF, and SPF mice received E-Mitter implant surgery followed by at least two weeks of recovery time. Double asterisk: Mice switched from NC to HF diet after D0 fecal collection. *GF* Germ-free; *SPF*  specific pathogen-free, *NC* normal chow, *HFD* high-fat diet, *LD*  12L:12D light–dark cycle, *DD* constant darkness, *RF*  restricted feeding manipulation. Created with BioRender.com (**B**) Relative abundances of microbial taxa from SPF and ex-GF mice on NC diet. Heatmap depicts relative abundance of each taxonomic feature for donor SPF mice (top row) and for both SPF and ex-GF mice on NC diet over all sampled time points throughout the study n = 8 for SPF mice at each time point, n = 5 for ex-GF mice at baseline, and n = 6 for ex-GF mice at all other time points. Several genera found in ex-GF mice that were not observed in the donor sample were presumably acquired from the environment post-conventionalization (for all 16S taxonomic features, see Table S1). Heatmap created using anvi’o v5.

To examine the sufficiency of the gut microbiome in driving circadian rhythms, GF mice were conventionalized by oral gavage with cecal contents from SPF donor mice, which effectively reconstituted most donor gut microbiota in the recipient animals (henceforth ‘ex-GF’ mice; 71%, or 33 ± 4.1, out of 47 donor genera restored), which persisted throughout the experiment (Fig. 5, Fig. S4, Table S1). Conventionalization caused most chronotypes of ex-GF mice to resemble those of SPF controls (Figs. 2, 3, 4). In the LD photocycle, LMA circadian amplitude (t_22_ = 1.759, P = 0.0925), LSP power (t_22_ = 0.034, P = 0.9729), activity counts (t_22_ = 1.197, P = 0.2439), onset precision (t_22_ = 1.876, P = 0.0739), onset time (t_22_ = 1.652, P = 0.1128), offset time (t_22_ = 1.264, P = 0.2195), and both α_LMA_ (t_22_ = 0.287, P = 0.7768) and α_Tb_ (t_20_ = 1.239, P = 0.2298) of ex-GF mice were restored to values indistinguishable from those of SPF controls. In DD, LMA amplitude (t_21_ = 0.668, P = 0.5117; Fig. 4F), LSP power (t_21_ = 1.587, P = 0.1274; Fig. 4F), circadian period (t_21_ = 1.353, P = 0.1903; Fig. 4B), α_LMA_ (t_21_ = 1.014, P = 0.3221; Fig. 4D) and α_Tb_ (t_20_ = 1.089, P = 0.2889; not illustrated) were likewise normalized by conventionalization. Conventionalization did not restore GF-associated changes in absolute T_b_ (SPF vs ex-GF, t_20_ = 2.447, P = 0.0238; Fig. 3C) in LD. In DD, ex-GF mice remained hypothermic relative to SPF controls (SPF vs ex-GF; daily, T_b_, t_20_ = 2.183, P = 0.0411; active (α) phase, t_20_ = 3.207, P = 0.0044; rest (ρ) phase, t_20_ = 1.079, P = 0.2933; Fig. 4G).

### The microbiome increases circadian responses to food but not light

**Figure 6:**
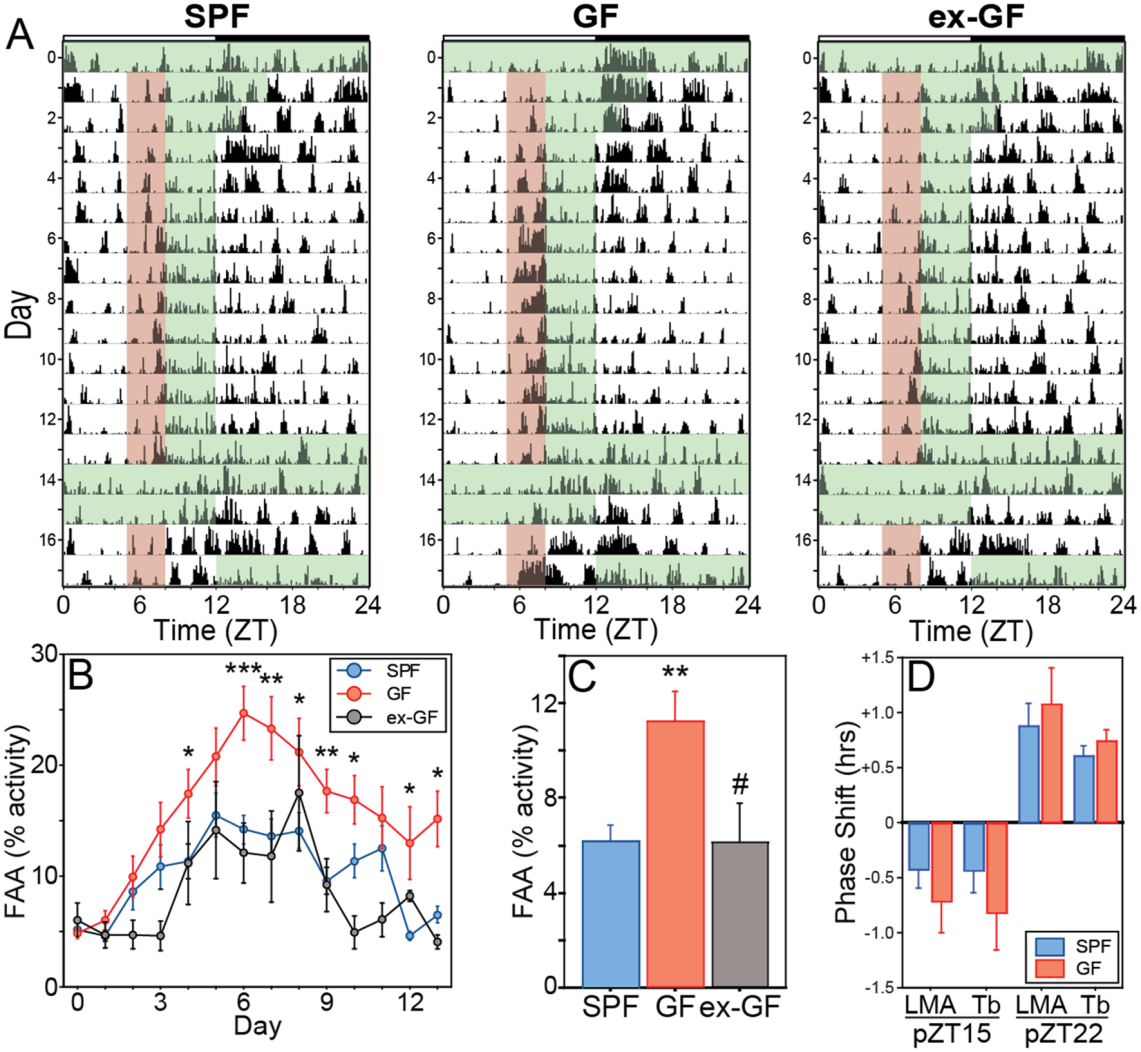
The microbiome facilitates entrainment to food, but does not affect circadian phase resetting responses to light. (**A**) Representative, single-plotted home-cage locomotor activity (LMA) records of SPF (left panel), GF (middle panel), and ex-GF mice (right panel) subjected to the restricted feeding (RF) paradigm. Green shaded regions indicate intervals when food was available. Red shading indicates the 3 h interval of activity coded as food anticipatory activity (FAA), which emerged over the course of several days during RF. During the last 48 h of RF, mice were food deprived to elicit re-emergence of FAA (retention probe trial). (**B**) Mean ± SEM FAA of SPF, GF and ex-GF mice subjected to RF expressed as a percentage of total daily activity during the 10–12 days of RF. (**C**) Mean + SEM FAA on the 2nd day of the retention probe trial. SPF: n = 18; GF: n = 16; ex-GF: n = 6 (**D**) Mean + SEM phase shift in the circadian LMA rhythm in response to 15 min light pulses at either projected ZT15 (‘pZT15’, left) or pZT22 (right). SPF: n = 10; GF: n = 8. *P < 0.05, **P < 0.01, ***P < 0.001 vs SPF mice; ^#^P < 0.05 v GF mice.

To further evaluate the role of host microbes on circadian behavior and responsiveness of the circadian network to environmental input, LMA and T_b_ responses were quantified in GF and SPF mice presented with multimodal stimuli capable of eliciting circadian phase resetting responses (*Zeitgebers*; see *Materials and Methods*). Circadian responses to non-photic cues were evaluated by challenging mice with a restricted feeding (RF) regimen, in which food was available for a limited interval (4 h) only during the light phase (Fig. 6A). In nocturnal rodents, RF initiates a reorganization of the circadian network, desynchronizing food-entrained and light-entrained circadian oscillators in the CNS and periphery^17^. Behavioral and thermoregulatory responses to RF include the emergence of fragmented activity and T_b_ rhythms: a novel component of daily activity (food anticipatory activity [FAA]) appears during the interval (3 h) immediately preceding food availability, and quantifies the magnitude of circadian entrainment to food^18^. FAA was greater in GF mice relative to SPF controls during the RF regimen (F_2,136_ = 7.384, P = 0.0152; Fig. 6B) and FAA was more persistent thereafter, indicated by a ~ two-fold increase in FAA during the retention probe trial after discontinuation of RF (t_32_ = 3.552, P = 0.0012; Fig. 6C). Conventionalization rendered FAA of ex-GF mice indistinguishable from that of SPF controls (t_22_ = 0.047, P = 0.9631; Fig. 6C).

Circadian responses to photic *Zeitgebers* were evaluated using: (1) discrete pulses of light delivered 3 h or 10 h into the subjective night (projected *Zeitgeber* Time [pZT] in constant darkness at pZT15 and pZT22), which elicit phase advances and delays, respectively, in the circadian pacemaker; and (2) a simulated 'jet lag' challenge consisting of a 6 h advance shift of the full LD cycle, which elicits re-entrainment. GF and SPF mice exhibited comparable delay (LMA, t_16_ = 0.948, P = 0.3571; T_b_, t_16_ = 1.059, P = 0.3053; Fig. 6D) and advance (LMA, t_16_ = 0.544, P = 0.5939; T_b_, t_16_ = 0.947, P = 0.3576; Fig. 6D) responses to acute light pulses at pZT15 and pZT22, respectively. Re-entrainment responses to simulated jet lag were analyzed separately for each replicate experiment due to a significant order effect (F_1,319_ = 69.318, P < 0.0001). The effect of microbial status on jet lag was inconsistent across the two cohorts: in Cohort 1, GF mice re-entrained LMA faster than SPF mice (F_1,176_ = 14.354; P = 0.0016; Fig. S5 A,B), while in Cohort 2 SPF and GF mice exhibited comparable re-entrainment responses (F_2,198_ = 0.625; P = 0.5464; Fig. S5 C,D). Ex-GF mice also did not differ in their re-entrainment responses compared to SPF or GF mice in Cohort 2 (F_2,198_ = 0.625; P = 0.5464; data not illustrated). In neither experiment did T_b_ re-entrainment responses differ among groups (Cohort 1, F_1,132_ = 0.416, P = 0.5312; Cohort 2, F_2,192_ = 1.386, P = 0.2786).

### Circadian effects of a high-fat diet persist absent host microbiota

A diet high in fat and simple sugar (HFD) profoundly alters gut microbial community membership, circadian taxonomic rhythms, and microbial metabolic activity, resulting in diet-induced obesity (DIO^9^). HFD also changes period of the circadian pacemaker, entrainment to external time cues, and internal phase relations among circadian clock genes in central and peripheral oscillators^19,20^. GF mice are refractory to DIO, but the extent to which microbial metabolism participates in the effects of HFD on organismal-level circadian rhythms in LMA and T_b_ has not been examined. Consistent with prior reports^8,9,21^, SPF mice fed HFD exhibited greater adiposity and liver mass compared to mice fed normal chow (NC), whereas GF mice fed HFD were resistant to DIO (Fig. S6). HFD also dramatically shifted microbial community membership (PERMANOVA, p ≤ 0.0005, Fig. S7 and Fig. S8A–C), with marked changes in abundance of taxa belonging to the families Streptococcaceae (genus *Lactococcus*), Erysipelotrichaceae (genus *Faecalibaculum*), and Verrucomicrobiaceae (genus *Akkermansia*) that persisted throughout the experiment (Fig. S9).

**Figure 7:**
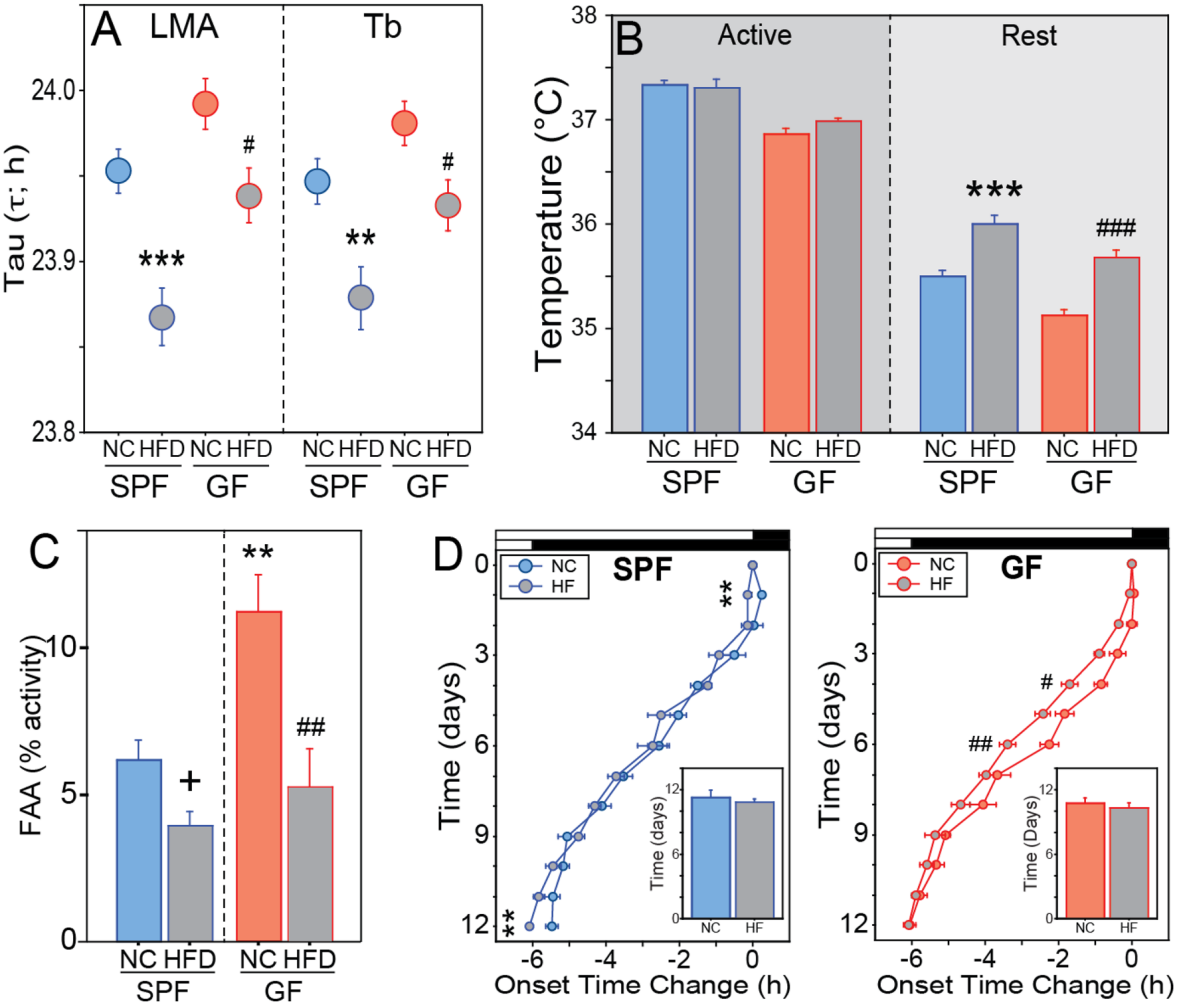
Diet high in fat and simple sugar (HFD) alters circadian activity, temperature rhythms, and behavioral responses to photic and non-photic *Zeitgebers*, independent of microbes. (**A**) Mean ± SEM period (in h) of the free running circadian rhythm of LMA (left panel) and T_b_ (right panel) of SPF and GF mice on NC or HFD and housed in DD. (**B**) Mean + SEM T_b_ during the subjective night (active phase; dark shading) and subjective day (inactive phase; lighter shading) portions of the circadian cycle of SPF and GF mice housed in DD and fed NC or HFD. (**C**) Mean + SEM food anticipatory activity (FAA) on the 2nd day of the retention probe trial of SPF and GF fed NC or HFD while subjected to the RF regimen. (**D**) Mean ± SEM activity onsets prior to (day 0) and following a 6 h advance shift of the photocycle of SPF (left panel) and GF (right panel) mice fed NC or HFD; insets: mean + SEM number of days required to re-entrain to the 6 h shift (see Supplementary Information and Methods for re-entrainment criterion). LMA, SPF-NC: n = 8–18; SPF-HFD: n = 7–8; GF-NC: n = 8–16; GF-HFD: n = 7. T_b_, SPF-NC: n = 18; SPF-HFD: n = 8; GF-NC: n = 15; GF-HFD: n = 7. ^+^P = 0.059, **P < 0.01, ***P < 0.001 v SPF-NC mice; ^#^P < 0.05, ^##^P < 0.01, ^###^P < 0.001 v GF-NC mice.

HFD altered the period of LMA and T_b_ circadian rhythms in DD, shortening circadian period in both SPF (τ_LMA_, t_24_ = 3.889, P = 0.0007; τ_Tb_ t_24_ = 2.837, P = 0.0084) and GF (τ_LMA_, t_21_ = 2.156, P = 0.0428; τ_Tb_, t_20_ = 2.241, P = 0.0365) mice alike (Fig. 7A; representative actograms Fig. S10). HFD markedly lengthened circadian α in GF mice (α_LMA_, t_21_ = 4.113, P = 0.0005; α_Tb_, t_20_ = 4.334, P = 0.0003; Fig. S11A,B) but had no such effect in SPF mice (α_LMA_, t_24_ = 0.462, P = 0.6481; α_Tb_, t_24_ = 1.381, P = 0.1801; Fig. S11B). Dietary shift to HFD likewise did not impart uniform changes in onset variability and rhythm amplitude in LD (Fig. S11B–E). However, HFD-induced daytime (rest phase) hyperthermia of 0.3–0.6 °C was observed, irrespective of microbial status or photocycle (DD: SPF-NC v -HF, t_24_ = 4.994, P < 0.0001; GF-NC v -HF, t_20_ = 6.523, P < 0.0001; LD: SPF-NC v -HF, t_24_ = 6.600, P < 0.0001; GF-NC v -HF, t_20_ = 2.858, P = 0.0097; Fig. 7B and Fig. S11F). The absence of microbes also did not prevent effects of HFD on circadian responses to *Zeitgeber* challenges: HFD inhibited FAA expression during retention trials in SPF (t_23_ = 1.987, P = 0.0590) and GF mice (t_21_ = 2.765, P = 0.0116; Fig. 7C). There was a main effect of HFD on accelerating re-entrainment to simulated jet lag (F_1,286_ = 6.599, P = 0.0163; Fig. 7D), with no main effect of microbial status (F_1,286_ = 0.2990, P = 0.2990; Fig. 7D) or interaction (F_1,286_ = 0.1960, P = 0.6616; Fig. 7D); but this was largely driven by HFD mice exhibiting sporadically advanced activity onsets on individual days during the process of re-entrainment to jet lag (Fig. 7).

## Discussion

Mice devoid of all microbes exhibited atypical circadian rhythms in activity and thermoregulation, including higher-amplitude rhythms, longer circadian period, and a strikingly greater responsiveness to the entraining effects of food. Reversal of these effects by conventionalization revealed a critical role for the concurrent activity of gut microbes, supporting the hypothesis that microbes contribute to the generation and entrainment of organismal-level circadian rhythms in multiple traits.

Behavioral and thermoregulatory rhythms of GF mice were atypical in a light–dark cycle (LD) and under conditions of constant darkness (DD). In LD, GF mice exhibited a shorter active phase, less activity during the rest phase, and substantially more precise daily activity onsets compared to mice harboring full microbial communities. The normalization of onset variance in GF mice upon release into DD suggests that some circadian effects of microbes may be restricted to rhythm entrainment processes rather than rhythm generation. In contrast, the marked compression of nocturnal activity among GF mice in LD conditions persisted in DD, suggesting these effects of microbial status on circadian oscillators that govern the duration of activity^22,23^ cannot be a result of changes in the perception of light (light intensity), nor can they solely reflect microbe-driven masking responses to light or darkness. Instead, these results indicate microbes impact the circadian pacemaker that generates overt circadian rhythms. Exposure to DD did, however, reveal a modestly significant effect of microbial status on a core feature of the circadian system: a longer period (τ) among GF mice. Moreover, most of the chronotypes evident in GF mice were eliminated by reintroduction of gut microbiota in ex-GF mice. Together, these data demonstrate that typical patterns of host endogenous circadian period and entrainment depend on the presence of a complex microbial community.

Compared to SPF mice, GF mice exhibited higher levels of entrainment to a non-photic *Zeitgeber* (food), but comparable levels of phase-resetting responses to photic *Zeitgebers* (light pulses at pZT15 and pZT22, 6 h phase shift). One critical feature of a circadian pacemaker is its ability to shift phase in response to environmental time cues. Timed restricted feeding (RF) paradigms have been used to uncouple, or desynchronize, normal phase relations among clocks in the circadian network; in response to RF, many peripheral circadian clocks abandon their previous phase and align with the timing of food appearance, whereas the clock(s) in the hypothalamic pacemaker remains phase-locked to the LD cycle^17,24–26^. Under RF conditions, the dissociation of daily activity into two components, one associated with the scotophase (nocturnal α) and another associated with food anticipation (food anticipatory activity; FAA), is inferred to reflect the activity of light-entrainable and food-entrainable components of the circadian system, respectively, which under conditions of ad libitum feeding are normally coherent and overlap in phase with one another at night^27^. The increased FAA among GF mice in the present experiments indicates that in the absence of microbial activity the overt expression of circadian clocks associated with food-related cues (i.e., food-entrainable oscillators; FEOs) is potentiated. This implies that one function of a normal complement of microbes that reside in and on the body may be to inhibit the independent expression of FEOs, or the ready fragmentation/dissociation of the circadian network into food- vs. light-entrainable components. In other words, the present data suggest that one function of the microbiome may be to promote phase coherence, or coupling, among the many oscillators within the host circadian network. Such a role would be consistent with recent observations that circadian oscillators in the brain and liver exhibit circadian phase-dependent shifts in clock gene expression in response to specific gut microbial metabolites, e.g., butyrate^8^.

In a comprehensive battery of circadian behavioral testing, the effects of a Western diet high in fat and sugar (HFD) on rhythms in LMA and T_b_ were widespread: mice on HFD exhibited shorter circadian period for both LMA and T_b_, lower rhythmic power, greater onset variability, overall faster jet-lag recovery, lower FAA and elevated daytime T_b_. In common with other reports^19,20^ HFD altered circadian τ (Fig. 7A), however the direction of this effect observed here was different, which may be attributable to the later age at which HFD was implemented and/or the later age at which mice were tested in the present report. The developmental stage at which diet manipulations are introduced may impact the magnitude of their effects on the circadian system, in common with other behavioral and physiological systems^28–30^. Notably, most circadian responses to HFD were evident in GF mice as well. These data also identified circadian effects of HFD unique to GF mice and not exhibited by SPF mice (e.g., α duration). These outcomes are consistent with the interpretation that gut microbes are not required for many of the host’s circadian behavioral and thermoregulatory responses to HFD. Microbes are, however, essential mediators of DIO and are necessary for normal, HFD-induced shifts in hepatic core circadian clock gene expression patterns^8^. Indeed, a mounting body of evidence indicates that intestinal microbial metabolites are necessary and sufficient mediators of HFD on many peripheral transcriptional and metabolic processes^31^. Consistent with previous reports, GF mice were protected from the metabolic consequences of HFD^8,32^. Together, the present data illustrate a novel and important distinction between effects of HFD on metabolic dysregulation and circadian clock gene rhythms in peripheral tissues, versus effects of HFD on overt rhythms in circadian behavior and thermoregulation: whereas microbes are both necessary and sufficient for HFD to induce metabolic disruption and to shift peripheral clock gene rhythms^8,33^, microbes may not be required for HFD to impact circadian rhythms in activity and T_b_ measured here. Although the present data cannot address whether specific microbial metabolites are sufficient to mediate effects of HFD on behavioral rhythms, these outcomes suggest that remediation of diet-induced disruptions in circadian behavioral rhythms may incorporate microbiome-independent mechanisms.

The present data replicate prior reports of global hypothermia in GF mice^34^, extending such observations to identify persistent hypothermia during both phases of the circadian cycle and a lower amplitude circadian T_b_ rhythm in GF mice. Whereas reintroduction of a semisynthetic microbial community at 6 weeks of age restored T_b_ amplitude in GF mice^34^, conventionalization of GF mice with SPF cecal contents in adulthood failed to do so (Fig. 3). In humans, the gut microbiome assembles and matures during the first 2–3 years of life^35^, and in mice gut microbes are required during critical windows of early development for normal maturation of immunological, neurological, and metabolic traits^36,37^. Assuming the complex microbiome used for conventionalization in the present work sufficiently restored levels of microbial complexity in ex-GF mice that were functionally equivalent to those of SPF controls (Fig. 5), its inadequacy in restoring euthermic T_b_ indicates the age of recolonization may factor critically in thermoregulation following conventionalization; early-life exposure to microbes may exert enduring effects on host thermoregulation and metabolism. To further explore sensitive periods to microbiome effects on host thermoregulation, future studies may conventionalize GF mice by introducing microbial communities either early (e.g., during the periweaning period) or later in life (e.g., 6 or 20 weeks of age).

Whereas the ex-GF condition establishes that microbes are sufficient to impact circadian phenotypes, given the longitudinal design of our study, one key limitation is that we have not yet linked specific microbial community members (or their functions) to circadian network dynamics; however, the experimental paradigms we describe here provide a guide for future studies to identify microbial contributions toward constitutive circadian network function in response to diverse environmental perturbations. In addition, these studies were performed using male mice. Given the well-documented sex differences in CRs^38^ and susceptibility to diet-induced obesity^39,40^, future studies should also explore germ-free and conventionally-raised female counterparts under identical experimental conditions outlined in this report. Finally, unlike their GF counterparts, SPF and ex-GF mice were not housed inside isolators during circadian measurements, thus we cannot determine whether, or the extent to which, housing conditions contribute to these phenotypes. The observation that several circadian traits were comparable between GF and either SPF or ex-GF mice suggests that housing conditions alone are not sufficient to alter circadian measures in these traits. Future studies using antibiotic-treatments to deplete gut microbiota may provide convergent evidence on this point.

Compared to SPF mice, GF mice in the present report exhibited higher-amplitude CRs, longer circadian periods, more precise (less variable) activity onsets in LD but not DD, and greater levels of FAA. Such an idiosyncratic constellation of chronotypes does not indicate that microbes uniformly enhance or impair host circadian rhythms. Greater onset variability is typically associated with an aging circadian system^41–44^, but the effects of microbial status on this measure were only evident in LD, leaving it unclear as to whether microbes affect pacemaker precision, light perception, or an interaction thereof. FAA reveals plasticity in circadian behavior: but the mere capacity for robust food entrainment may confer benefits or decrements, depending on the relation between the timing of food and the organism’s temporal niche (i.e., nocturnal vs. diurnal)^45,46^.

In summary, microbial communities within the host influence both generation and entrainment of normal circadian rhythms in LMA and T_b_. Changes in the waveform of entrained and free-running circadian rhythms, along with more prominent rhythm bifurcation in the face of a non-photic *Zeitgeber* (i.e., food) in GF mice, suggest that one possible function of the microbiome may be to facilitate coupling among circadian oscillators distributed throughout the body, stabilizing host circadian networks in the face of environmental challenges. Reversal of the GF chronotype after conventionalization indicates that the normal expression of circadian rhythms in adulthood is dependent on the contemporaneous activity of the microbiome. This stands in contrast to body temperature, which appears persistently and irreversibly reduced in mice deprived of microbes early in life, and effects of HFD on behavior, many of which manifested independent of microbes.

## Materials and methods

### Animals

Male C57Bl6/J specific pathogen free (SPF) mice from Jackson Laboratories were maintained in the University of Chicago animal vivarium under conventional housing conditions. Male C57Bl/6 germ-free (GF) mice were bred and maintained in sterile, flexible film isolators (Class Biologically Clean, Madison, WI); sterility was confirmed via analysis of bi-weekly fecal collections using culture-dependent and independent assays. GF mice were removed from sterile isolators and conventionalized (ex-GF) via gavage inoculation with 150uL of cecal slurry (100 mg/ml, in sterile PBS) obtained from age-matched male SPF mice 6 weeks prior to data collection as previously described^8^. Light cycles were 12L:12D (lights off at 06:00 PM CST) unless otherwise noted. Temperature and relative humidity were 19 ± 2 °C and 53 ± 10%, respectively. Autoclaved pine shavings were provided to all mice and autoclaved water was freely available. Mice were provided with either autoclaved normal rodent chow (NC; LabDiet 5k67) or an irradiated semi-purified high-fat diet containing 37.5% anhydrous milk fat (HFD; Envigo Harlan Teklad TD.97222 customized diet). Mice were fed ad libitum except during RF tests. All experiments were approved by the Institutional Animal Care and Use Committee (IACUC) of The University of Chicago and were performed in accordance with relevant guidelines and recommendations, including the guide for the care and use of laboratory animals. The University of Chicago maintains an AAALAC, Int., accredited animal care and use program and PHS assurance with OLAW. The reporting follows the recommendations in the ARRIVE 2.0 guidelines. Euthanasia was performed in accordance with the AVMA guidelines on euthanasia and approved by the University of Chicago IACUC.

Experiments were conducted in two cohorts (Fig. 1). Mice in Cohort 1 were fed normal chow (SPF-NC = 10, GF-NC = 8). Mice in cohort 1 were 11 to 18 weeks of age at the start of the 12:12 light:dark cycle data collection. Mice in Cohort 2 were randomly assigned to one of two diets and fed either normal chow (SPF-NC = 8, GF-NC = 8, ex-GF-NC = 6) or HFD (SPF-HFD = 8, GF-HFD; n = 8). Mice in cohort 2 were 17 to 24 weeks of age at the start of the 12:12 light–dark cycle data collection. A recent technical report describing the use of infrared beams to monitor mouse LMA in sterile isolators did not identify a main effect of age on total daily activity across a range spanning from 4 weeks to 8 months of age^47^. Telemetry data as well as body and organ weights were collected and analyzed from both cohorts. However, stool samples for 16S rRNA gene amplicon sequencing was performed solely from Cohort 2 mice.

### Surgical procedures

Pre-calibrated radio-telemetric transmitters (G2 E-Mitters; Starr Life Sciences; Oakmont, PA, USA) which transmit locomotor activity (LMA) and core body temperature (T_b_) were implanted i.p. under surgical anesthesia (dexdomitor, 1 mg/kg; ketamine, 75 mg/kg). Identical surgical procedures were performed inside the sterile environment for GF and ex-GF mice, and under aseptic conditions for SPF mice^32^. The anti-sedative atipamezole (5 mg/kg) was administered after surgery, and buprenorphine (0.1 mg/kg, s.c.) analgesic was administered immediately after surgery and at 12 h intervals thereafter for 48 h. Receiver boards (ER-4000 Energizer/Receiver; Starr Life Sciences) positioned under animal cages acquired T_b_ and LMA data at 1 min intervals using VitalView software (Starr Life Sciences). This methodology permitted measurement of LMA and T_b_ over indefinite time spans (i.e., multiple months) with sampling intervals sufficient for Fourier-based time series analysis of circadian activity and temperature rhythms, in contrast with other efforts^32,47,48^ which have been limited to brief intervals (≤ 5 days) and/or aggregate measures of total activity binned hourly or daily, inadequate for the quantitative evaluation of circadian behavioral rhythms.

### Circadian manipulations

LMA and T_b_ were recorded continuously prior to and during manipulations of the environmental photocycle (LD cycle) and food availability. Experiments were conducted in two cohorts (Fig. 1). Cohort 1’s order of circadian manipulations: entrainment to 12:12 LD cycle, jet lag, pZT15 light pulse, pZT22 light pulse, constant darkness, food entrainment. Cohort 2’s order of circadian manipulations: entrainment 12:12 LD cycle, constant darkness, jet lag, and food entrainment. Housing in 12L:12D allowed evaluation of steady-state entrainment to the LD cycle. Circadian shifts in response to simulated jet-lag were evaluated via a 6 h phase advance of the 12L:12D photocycle, which was accomplished in a single cycle with an advance in the onset of the dark phase. Phase shifting responses to discrete 15 min light pulses were performed after 48 h in DD, using an Aschoff Type II paradigm^49^. Circadian entrainment to food was quantified as food anticipatory activity (FAA) during which access to food was limited to a 4 h interval of the light phase, and during a 48 h episode of total food deprivation after the discontinuation of this restricted feeding schedule (RF).

### Circadian analyses

Circadian LMA and T_b_ data were collected in 1 min bins using VitalView and converted for analysis in Clocklab 6.1 software (Actimetrics, Wilmette, IL USA). To avoid arousal artifacts resulting from bi-weekly cage changing, analyses of behavior during entrainment to LD and exposure to DD were performed on 12 and 11 days of telemetry data, respectively. For each mouse, onset variability was calculated as the standard deviation (SD) of the 11–12 consecutive LMA and T_b_ onsets and offsets. Circadian onsets (of activity and of the circadian T_b_ rise/decline) and offsets were calculated using automated modules within Clocklab, with manual correction of erroneous onset/offset designations performed by an experimenter blind to treatment condition. Circadian alpha (α) was calculated as the interval between onset and offset. Total and phase-specific activity counts and mean T_b_ were calculated using the Activity Profile function in Clocklab. In LD, onset error was quantified as the standard deviation of consecutive onset times. In DD, onset error for free-running rhythms was quantified as the square-root averaged squared distance of data points deviating from the onset tau regression line. Circadian period (τ) was calculated as the slope of a regression line on successive circadian activity/T_b_ onsets. Amplitude of the circadian LMA and T_b_ waveforms was calculated using a non-parametric calculation based on a ratio comparing average activity during the intervals of greatest and least daily activity (NPCRA module, ClockLab^50^). Power of the circadian waveform was calculated as PN values using a Lomb-Scargle periodogram (LSP) analysis (ClockLab; threshold: P = 0.001). The Lomb-Scargle is a Fourier-based method which uses a least squares approach to determine the sinusoid whose period best fits the timeseries data. Power in the LSP calculation then refers to a measure of "goodness of fit" and a difference in power at the same dominant period reflects a difference in how well the underlying signal is modeled by a sinusoid of that period^51^.

Circadian phase shifting responses to light were quantified using an Aschoff Type II protocol^49^. Briefly, mice were transferred to DD, which eliminates masking effects of the light–dark cycle; after 2 circadian cycles (> 48 h) in DD delaying or advancing pulses of light (overhead light, ~ 400 lx, 15 min) were administered at projected ZT15 (‘pZT15’) or pZT22, respectively, after which time mice remained in DD for an additional 11 days. Phase shifts were calculated as the difference (in h) between the time of activity onset on the day of the light pulse, and an extrapolated regression line on 6–7 activity onsets of the post-pulse free-running rhythm, omitting 3 post-pulse transient cycles.

Adaptation to the 6 h phase advance of the full LD cycle (simulated jet lag) was quantified as the number of days required to shift activity onset by 6 ± 0.3 h (equivalent to 95% of the shift magnitude)^44,52^. Re-entrainment was also calculated using the midpoint of locomotor activity, which correlated with results using activity midpoint (*r* = 0.96, P < 0.0001; data not illustrated). To examine entrainment to food as a *Zeitgeber*, mice in LD were trained on a RF schedule. On the first day of RF, food was removed at the beginning of the light phase (ZT 0) and restored for 8 h (from ZT8 to ZT16) spanning 4 h of the light and dark phases to facilitate adaptation to the new feeding regimen. On the second day of RF, food was available from ZT8 to ZT14, spanning 2 h of the dark phase. For the remainder of the RF interval, food was available only during the light phase, from ZT8 until ZT12. RF lasted for 10 days in Cohort 1 and for 12 days in Cohort 2. At the end of RF training, food was available ad libitum for 2 days. All food was then removed completely for 48 h to assess retention of food-related circadian entrainment (FAA). By convention, FAA was recorded as the proportion of daily activity occurring during the 3 h immediately prior to the actual (training days) or expected (retention trial days) time of food delivery (FAA).

### 16S rRNA gene amplicon sequencing and analyses

Donor cecal contents used for conventionalization of ex-GF mice were stored at −80 °C until DNA was extracted. Fecal samples from SPF mice on NC and HFD and from ex-GF mice on NC diet were collected at baseline (~ 6 weeks after implant surgery, day 0) when all mice were still on NC; on experiment day 18 (~ 2 weeks after diet switch for SPF HFD-fed mice, D18); and on days 67 and 88 (immediately prior to, and after, the RF manipulation, D67 & D88). DNA extraction from fecal pellets and donor gavage solution was performed as previously described^53–55^, followed by amplification of the V4–V5 region of the 16S rRNA gene via standard protocols^56^, and DNA sequencing was performed at the High-Throughput Genome Analysis Core at Argonne National Laboratory from mice in Cohort 2. Sequences were demultiplexed, trimmed, filtered, and denoised using the QIIME2 toolkit and DADA2^57,58^ and mapped to the SILVA reference database at 97% sequence identity^59^; phylogenies were built with FastTree 2^60^.

### Statistical analyses

The sample size for Cohort 1 was adopted from previous work examining the effects of diet on circadian rhythms^19^. Cohort 2 sample sizes were determined based on Cohort 1 data via power calculation to detect differences between groups at 0.05 alpha, 0.80 power, and Cohen d’s effect sizes using python (3.8.3) package statsmodels (v0.13.0). 16S rRNA gene sequencing data analyses were performed in R using the phyloseq and vegan packages^61,62^, and Bray–Curtis beta diversity dissimilarity matrices were computed. PERMANOVA (permutational multivariate analysis of variance) and PERMDISP (homogeneity of multivariate dispersions) were performed^63^ followed by visualization in either R or anvi’o (v5)^64^. Analyses of variance (ANOVAs) were performed on LMA and T_b_ data; where warranted by a significant *F* statistic, two-tailed t-tests were performed (StatView; SAS Institute, Cary, NC). In order to permit parametric statistical analyses (e.g., ANOVA, t-test), behavioral datapoints that fell outside ± 3 SD from the sample mean (encompassing 0.3% of data) were classified as outliers and were omitted from statistical analyses^65^. Differences were considered significant if P ≤ 0.05. Experiments were conducted in two cohorts; LMA and T_b_ data were collapsed across both cohorts for statistical analyses if no significant effect of cohort was identified, which occurred in all cases except for responses to the jet lag manipulation (F_1,319_ = 69.318; P < 0.0001), which was therefore evaluated separately for each cohort.

## Supplementary Information

Supplementary Information.

## Data Availability

16S rRNA gene amplicon sequencing data has been deposited at NCBI SRA under BioProject accession number PRJNA664258. All additional data are available in the main text or the supplementary materials.
