# Supplementary material for: Atypical behavioral and thermoregulatory circadian rhythms in mice lacking a microbiome
Source: Sci Rep. 2022 Aug 25;12:14491. doi: 10.1038/s41598-022-18291-9 (PMC9411200; doi:10.1038/s41598-022-18291-9)
Supplement: Supplementary file 1 — Supplementary Information. [file 41598_2022_18291_MOESM1_ESM.docx]

Supplementary Materials for

**Atypical behavioral and thermoregulatory circadian rhythms in mice lacking a microbiome**

Vanessa A. Leone*, Kenneth G. Onishi*, Megan Kennedy, Jonathan P. Riggle, Joseph F. Pierre, Andrew C. Maneval, Melanie N. Spedale, Betty R. Theriault, Eugene B. Chang, Brian J. Prendergast

*Corresponding authors. Email: [valeone@wisc.edu](mailto:valeone@wisc.edu) and/or [kgonishi@uchicago.edu](mailto:kgonishi@uchicago.edu)

**This PDF file includes:**

Figs. S1 to S11

Table S1

**Fig. S1**


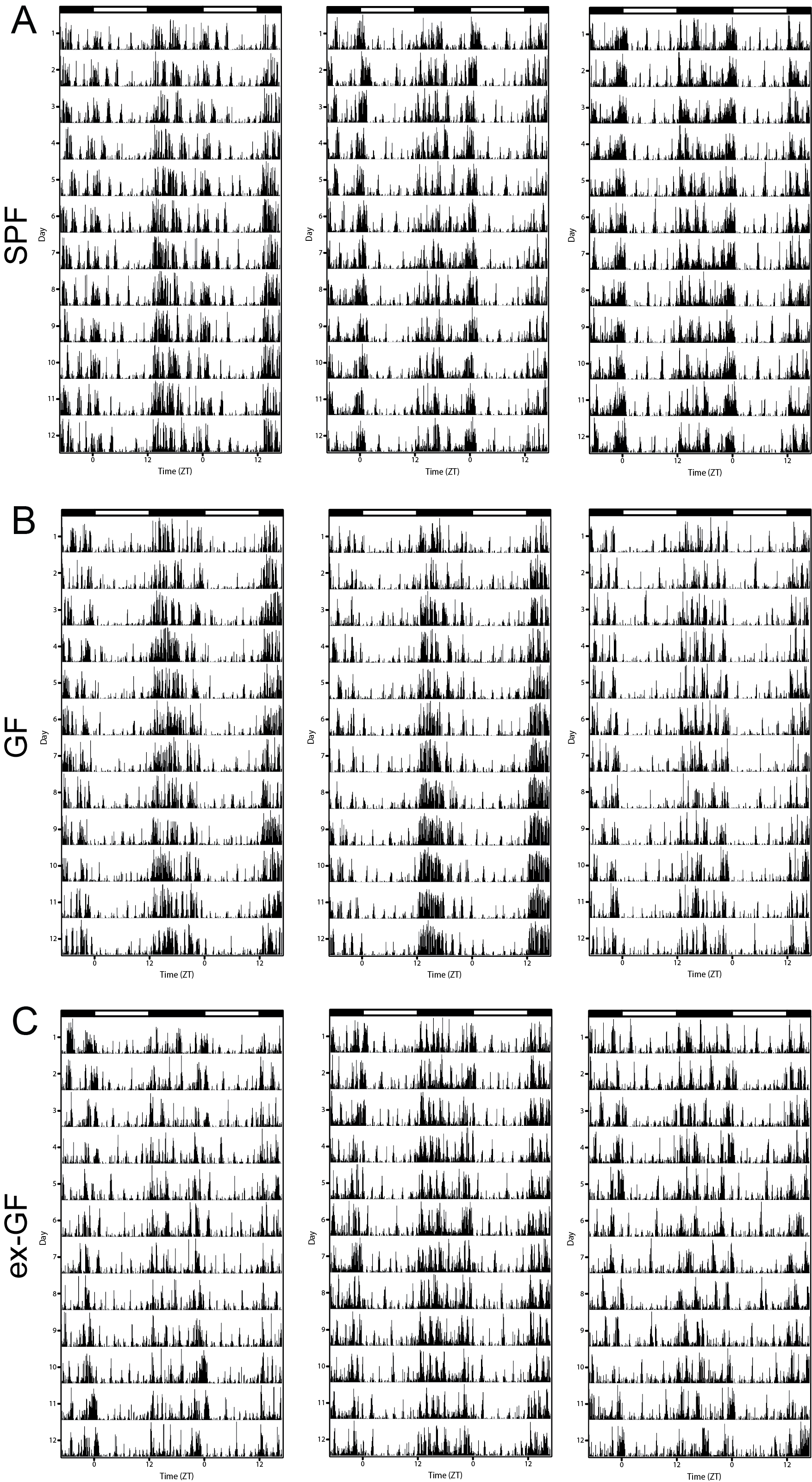


**Figure S1.** **Activity (LMA) and records of SPF, GF and ex-GF mice in a light:dark cycle.** Representative, double-plotted home cage activity (LMA) records of (A) SPF, (B) GF, and (C) ex-GF mice housed in a 12L:12D photocycle (LD). Time is indicated on the horizontal axis of each actogram, along with light (white) and dark (black) phases of the LD photocycle.

**Fig. S2**


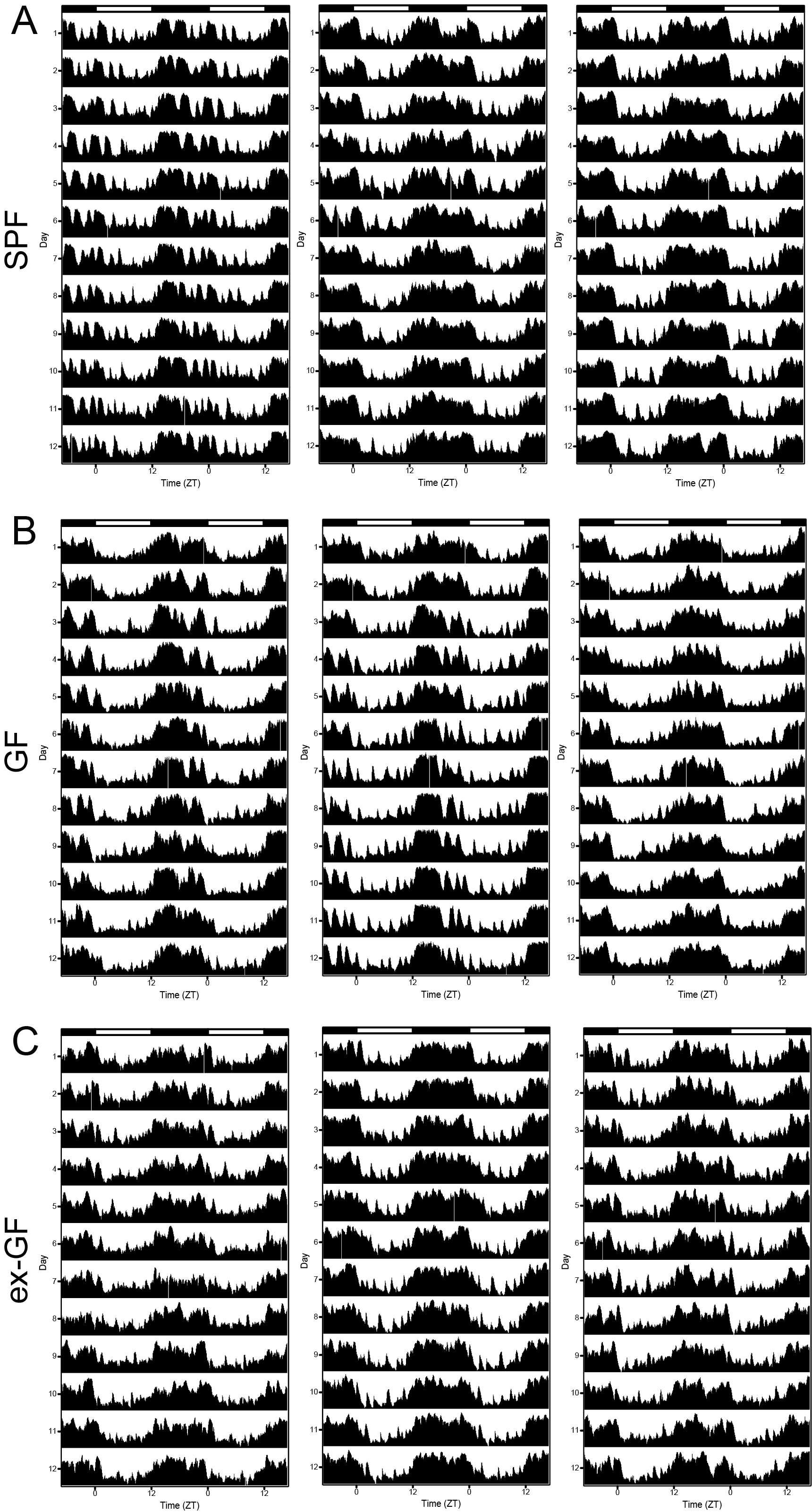


**Figure S2.** **Body temperature (T_b_) records of SPF, GF and ex-GF mice in a light:dark cycle.** Representative, double-plotted body temperature records of (A) SPF, (B) GF, and (C) ex-GF mice housed in a 12L:12D photocycle (LD). Time is indicated on the horizontal axis of each actogram, along with light (white) and dark (black) phases of the LD photocycle.

Fig. S3


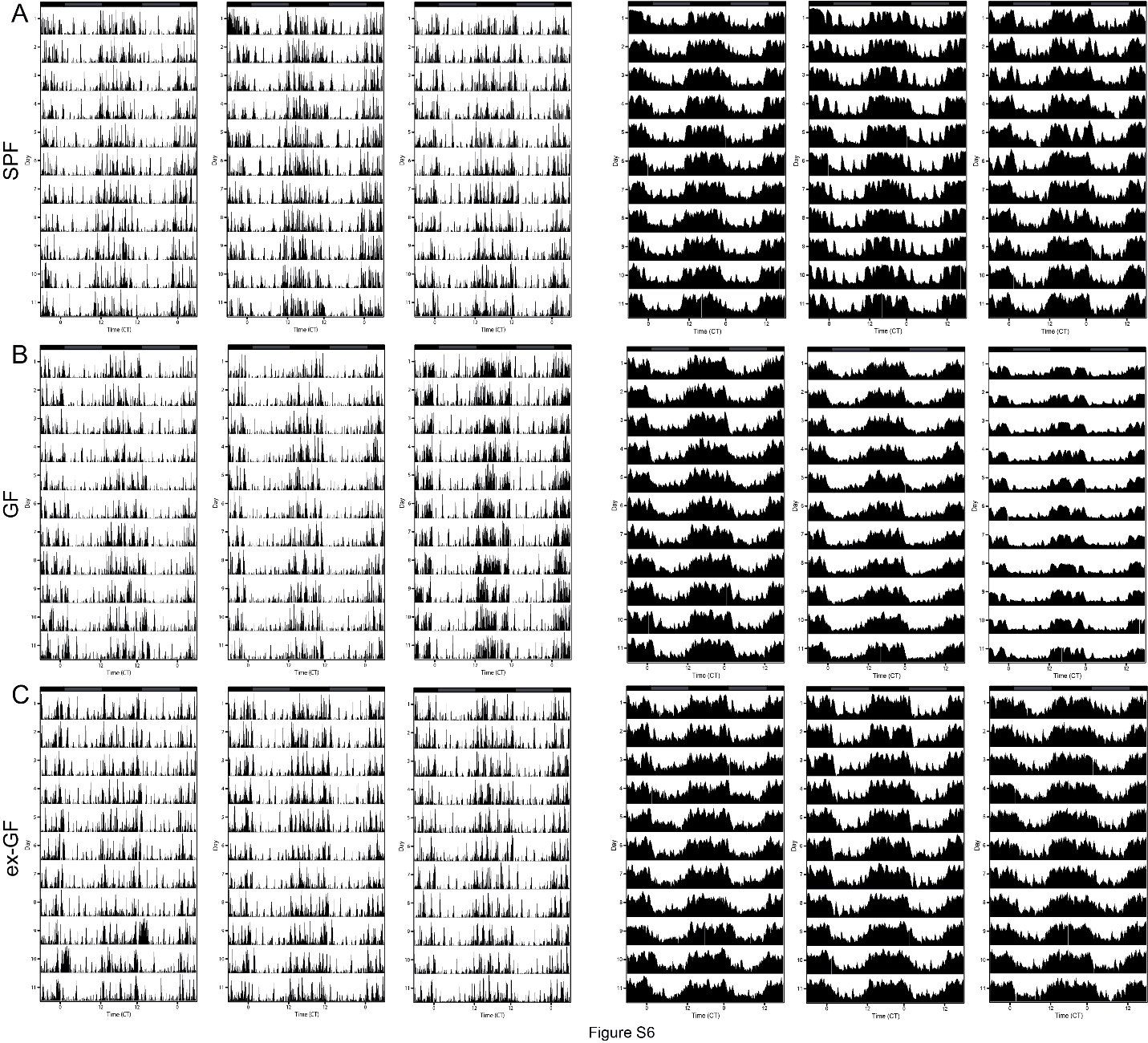


Figure S3. Activity (LMA) and temperature (T_b_) records of SPF, GF and ex-GF mice in continuous darkness. Representative, double-plotted home cage activity (LMA, left panels) and body temperature (T_b,_ right panels) records of (A) SPF, (B) GF, and (C) ex-GF mice housed in the DD photocycle. Circadian time (CT) is indicated on the horizontal axis of each actogram.

Fig. S4


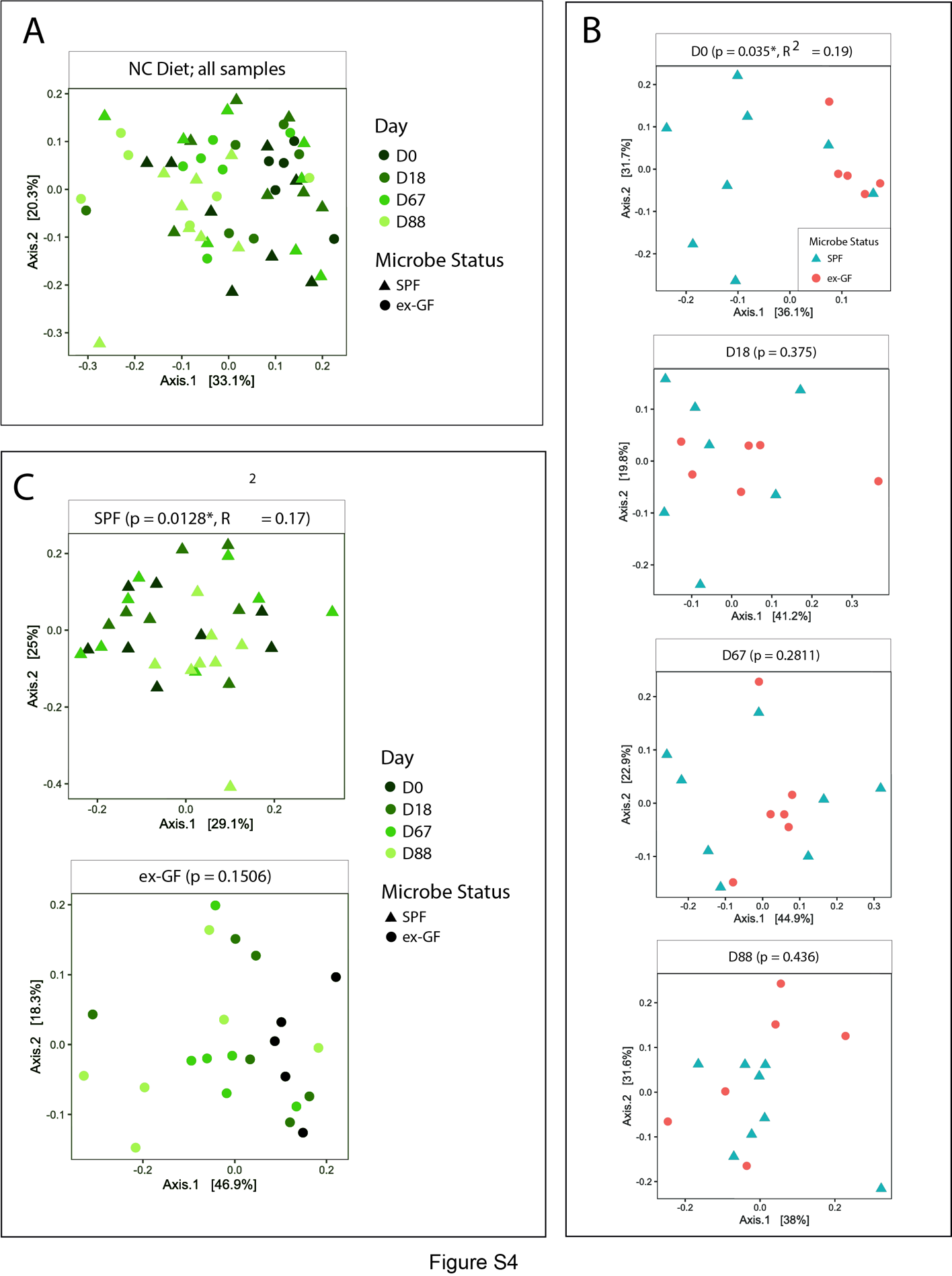


Figure S4. Principal coordinate analysis of gut microbial communities from SPF and ex-GF mice on NC diet. Taxonomic features were grouped at the genus level and samples were analyzed by Bray-Curtis dissimilarity. (A) Adonis PERMANOVA analyses were performed on data from all mice on NC diet to identify an effect of Microbe Status (p = 0.0147, R^2^ = 0.04) and Day (p = 0.0032, R^2^ = 0.12). (B) Adonis PERMANOVA analyses were performed within each time point to explore differences between ex-GF and SPF mice on NC diet at baseline, *day 0* (D0), *day* 18 (D18), *day 67* (D67)*,* and *day 88* (D88). SPF and ex-GF mice were significantly different at D0 (PERMANOVA, p=0.035), although this potential effect cannot be disentangled from the significant difference in variance between these communities at baseline (PERMDISP, p=0.0119); ex-GF and SPF mice were indistinguishable by D18 (PERMANOVA, p=0.375) and at all subsequent time points. (C) Adonis PERMANOVA analyses were performed within subsets of SPF or ex-GF mice on NC diet to explore differences in microbial composition across time points. Despite indistinguishable community membership between ex-GF and SPF mice by D18, SPF mice exhibited a significant shift across time (p = 0.0128, R^2^ = 0.17), which was not observed in ex-GF mice.

Fig. S5


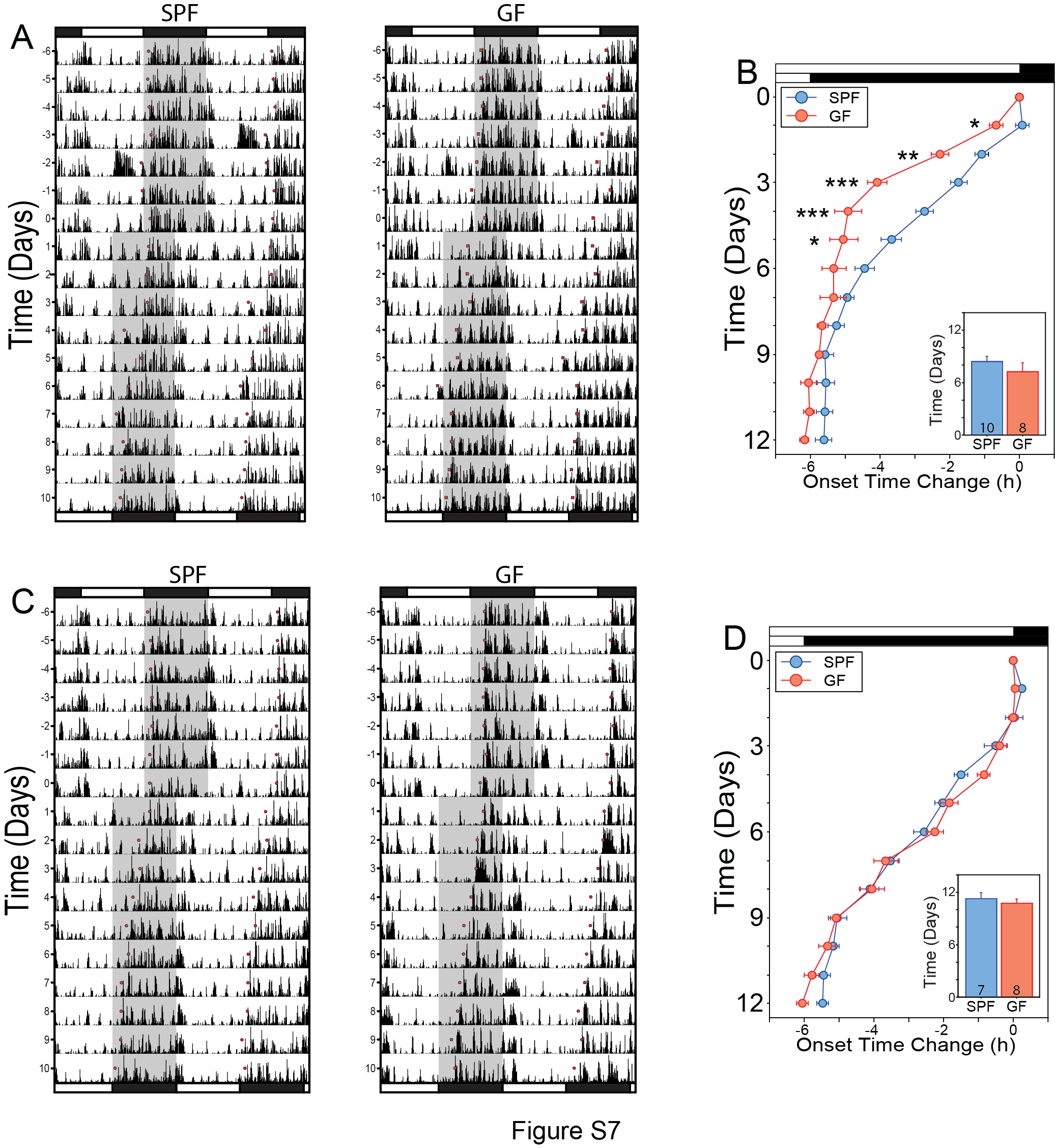


Figure S5. Effects of commensal microbes on re-entrainment to experimentally simulated ‘jet lag’. Data are depicted separately for the two experimental replication cohorts. Cohort 1: panels A-B; Cohort 2: panels C-D. Representative, double-plotted home cage activity (LMA) records of SPF (left record) and GF (right record) mice housed in a 12L:12D photocycle (LD) which was phase-advanced by 6 h via a shortening of the light phase on *day 0* (ordinate axis). Time is indicated on the top abscissa, along with light (white) and dark (black) phases of the LD photocycle prior to the phase shift; the new (post-shift) light-dark cycle is depicted on the bottom abscissa. (B,D) Mean ±SEM activity onsets of SPF and GF mice immediately prior to (*day 0*) and following a 6 h advance shift of the photocycle; inset: mean +SEM number of days required to re-entrain to the 6 h shift for each cohort of the experiment (see *SI Materials and Methods* for re-entrainment criterion). *P<0.05, **P<0.01, ***P<0.001 v corresponding SPF value.

Fig. S6


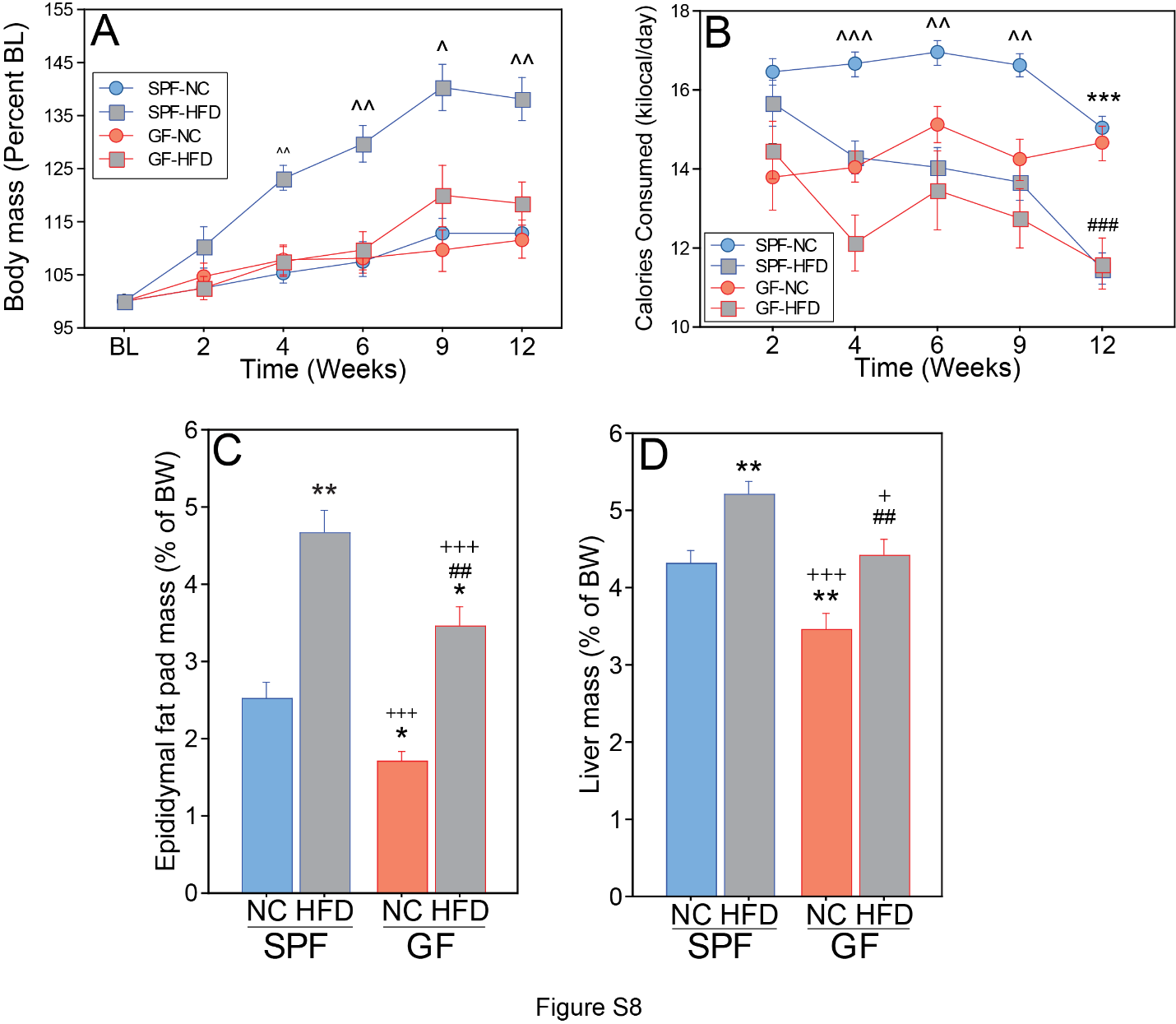


Figure S6. Somatic, ingestive and physiological responses to high fat diet (HFD) depend on presence of microbes. (A) Mean ±SEM body masses indicate that SPF-HFD mice gained more weight compared to all other groups over the course of the experiment. Body mass did not differ from baseline between SPF, GF or ex-GF mice during the observation interval (F_2,95_=0.02; P>0.90). (B) Mean ±SEM calorie intake data indicate that SPF-NC and GF-NC mice consumed more calories than SPF-HFD and GF-HFD, respectively. SPF mice consumed more kilocalories as compared to GF mice throughout the observation period (F_2,76_=13.8; P<0.0001). (C) Mean +SEM epididymal fat, and (D) liver mass data indicate that the magnitude of HFD-induced increases in adiposity and hepatic hypertrophy were attenuated in GF mice. When normalized to terminal body mass, GF mice exhibited significantly lower epididymal fat pad and liver masses relative to those of SPF mice (P<0.01, P<0.01, respectively), and ex-GF mice were comparable to SPF mice (P>0.40; P>0.80; respectively). SPF-NC: n=13; SPF-HFD: n=8; GF-NC: n=12; GF-HFD: n=7. ^P<0.05, ^^P<0.01 vs all other groups; **P<0.01, ***P<0.001 v SPF-NC; #P<0.05, ###P<0.001 v GF-NC; +P<0.05, +++P<0.001 v SPF-HFD.

Fig. S7


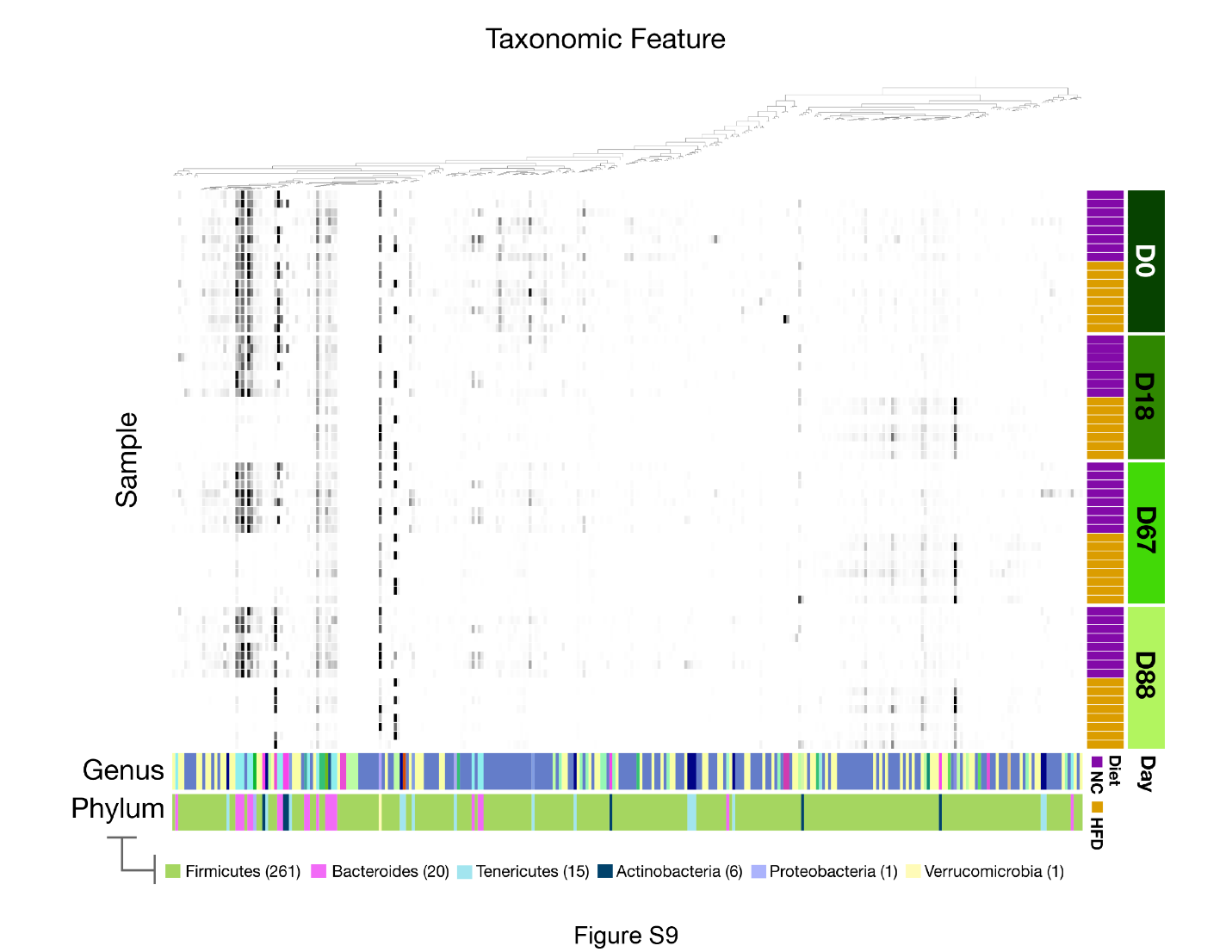


Figure S7. Relative abundances of microbial taxa from SPF mice in response to HFD. Heatmap depicts relative abundance of each taxonomic feature over all sampled time points for SPF mice on NC or HF diet. n=8 for each diet at each time point. Mice were maintained on NC through baseline, at which time they remained on NC or were switched to HFD for all remaining time points. Heatmap prepared using anvi’o (v5).

Fig. S8


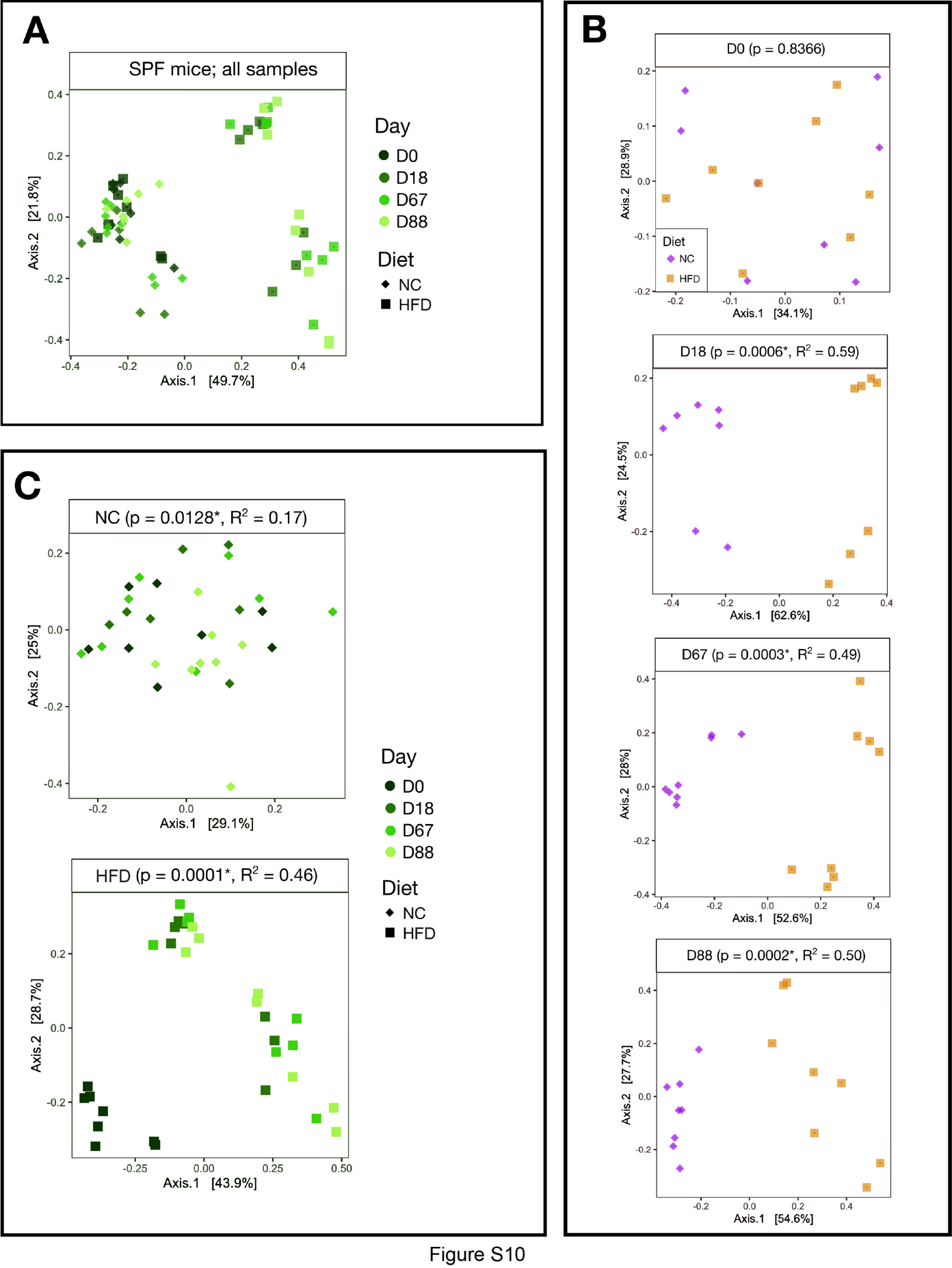


Figure S8. Principal coordinate analysis of gut microbial communities from SPF mice fed NC vs HFD. Taxonomic features were grouped at the genus level and samples were analyzed using Bray-Curtis dissimilarity. (A) Adonis PERMANOVA analyses were performed on data from all SPF mice to identify an effect of both diet (P= 0.0001, R^2^= 0.28) and day (P= 0.0001, R^2^= 0.16). (B) Adonis PERMANOVA analyses were performed within each time point to explore differences between SPF mice on NC or HFD at *day 0* (D0), *day* 18 (D18), *day 67* (D67)*,* and *day 88* (D88). All mice were indistinguishable at baseline, before dietary shifts were implemented, but on D18, mice on NC and HFD became significantly different and remained so for all remaining time points (P= 0.0006, R^2^= 0.59). (C) Adonis PERMANOVA analyses were performed within dietary treatments to explore differences in microbial composition across time points. Mice on both NC and HFD diet were significantly different across time points (NC: P= 0.0128, R^2^= 0.17, HFD: P= 0.0001, R^2^= 0.46).

Fig. S9


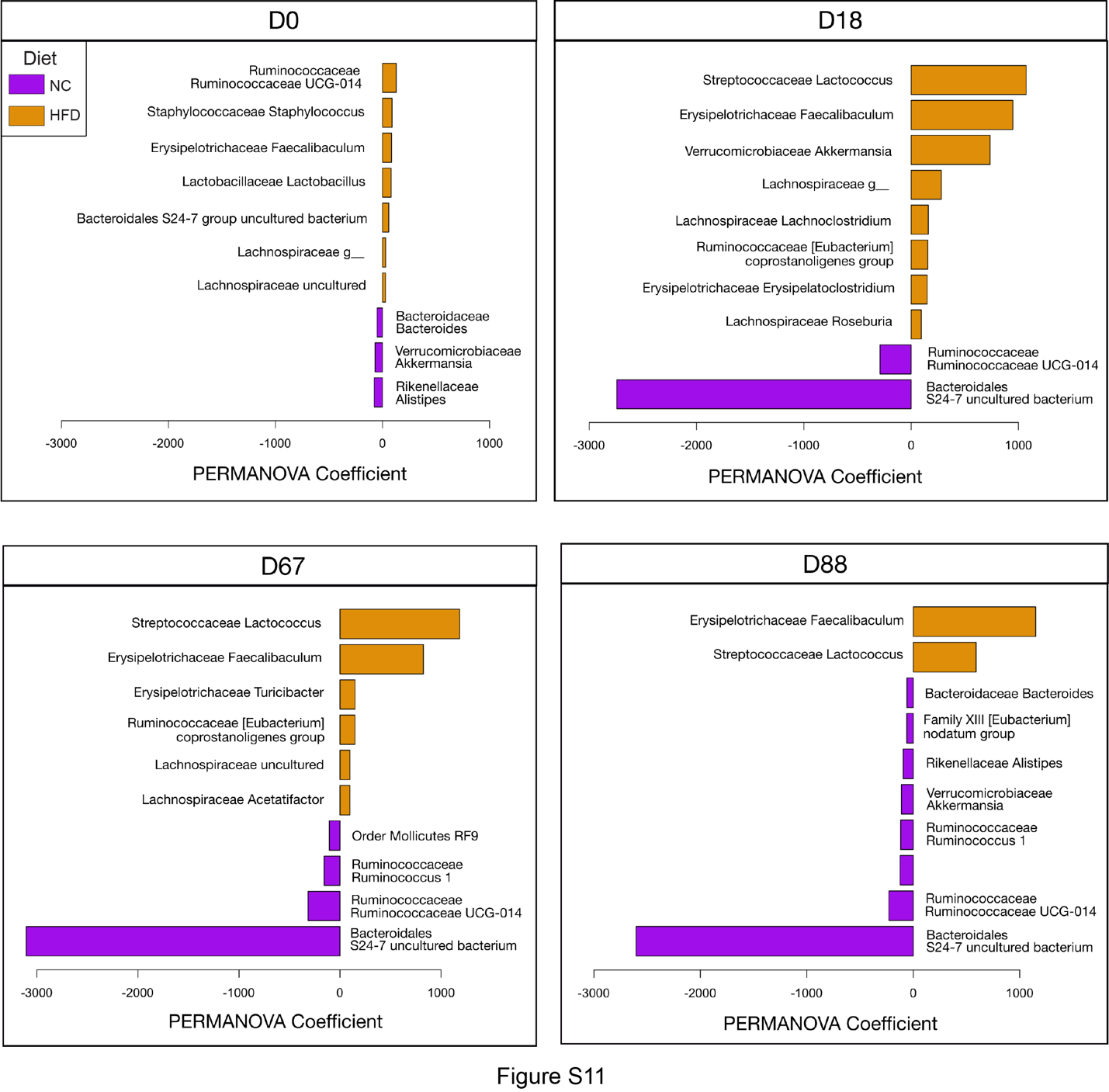


Figure S9. Taxa enriched in SPF mice on NC v HFD. Differences in gut microbial composition across NC and HF mice at time points *day 0* (D0), *day* 18 (D18), *day 67* (D67)*,* and *day 88* (D88) were driven largely by differential abundances of the families Streptococcaceae (genus *Lactococcus*), Erysipelotrichaceae (genus *Faecalibaculum*), and Bacteroidales (*S24-7*).

Fig. S10


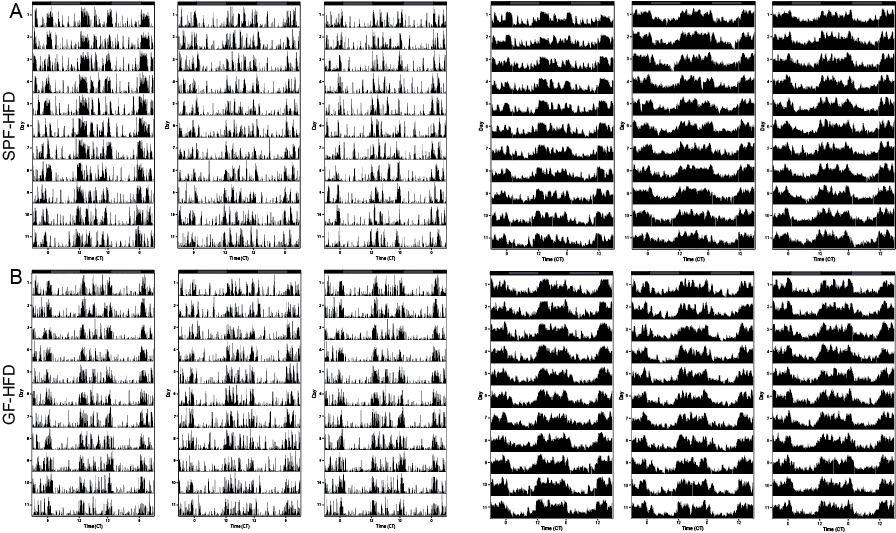
Figure S10. Activity (LMA) and temperature (T_b_) records of SPF and GF mice fed high fat diet in continuous darkness. Representative, double-plotted home cage activity (LMA, left panels) and body temperature (T_b_, right panels) records of (A) SPF-HFD and (B) GF-HFD mice housed in the DD photocycle. Circadian time (CT) is indicated on the horizontal axis of each actogram.

­­
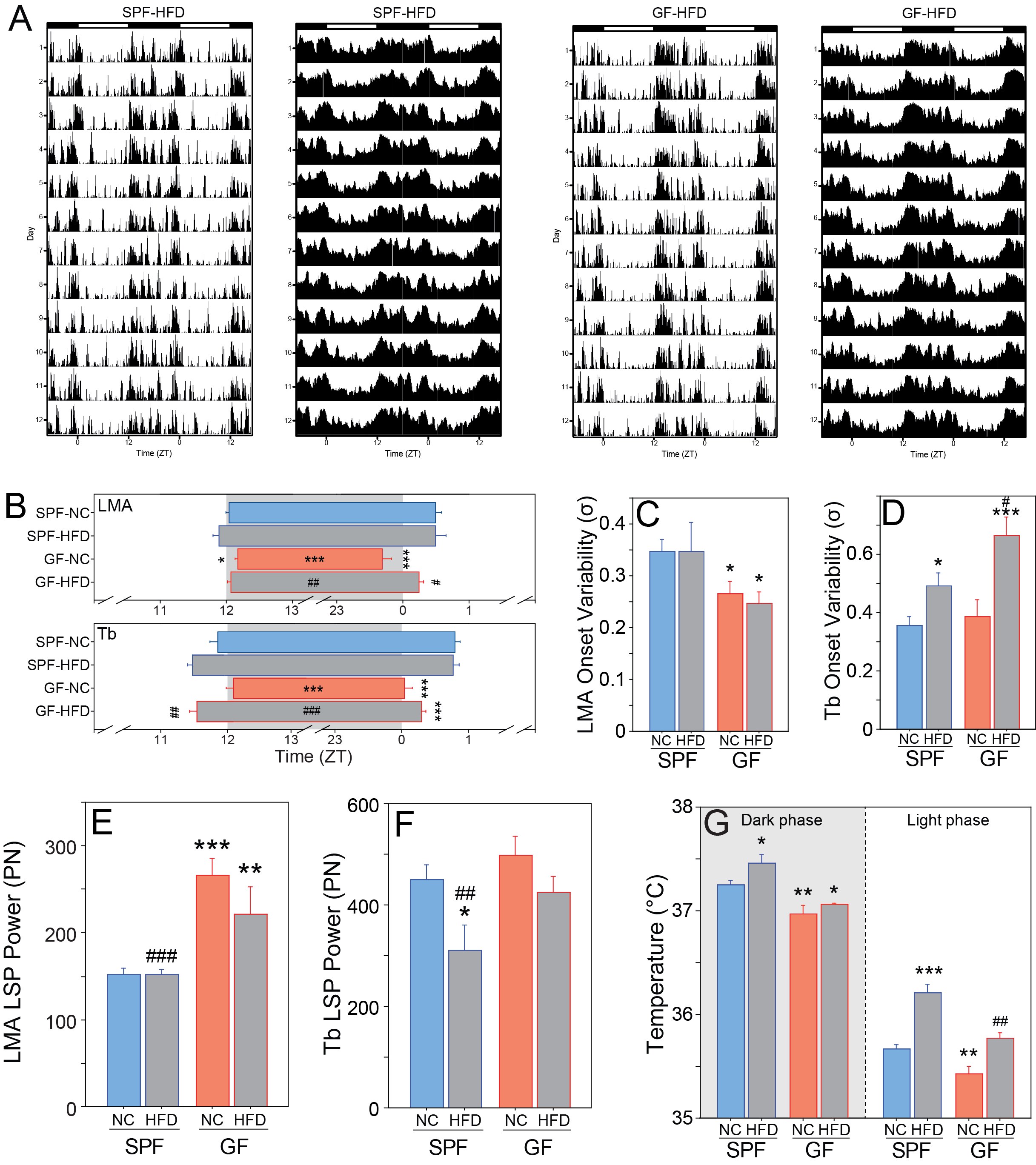


**Figure S11.** **Circadian LMA and T_b_ rhythms in SPF and GF mice on HFD diet in a 12:12 light:dark cycle.** (A) Representative, double-plotted home cage locomotor activity (LMA; first and third panels) and core body temperature (T_b_; second and fourth panels) records of SPF (left panels) and GF (left panels) mice housed in a 12L:12D photocycle (LD). (B) Mean +SEM onset and offset of daily locomotor activity (top panel) and beginning and end of nightly elevated T_b_ (bottom panel) of SPF and GF mice fed normal chow (NC) or a high fat diet (HFD) and housed in the LD photocycle (onset and offset are represented by the beginning and end of each bar, respectively). Mean +SEM variability in (C) activity (LMA) onsets and (D) the variability of the nocturnal T_b_ rise of SPF and GF mice housed in LD and provided with HFD or maintained on a NC diet. Mean +SEM circadian power (PN values, Lomb Scargle periodogram) of circadian rhythms in (E) LMA and (F) T_b_ of SPF and GF mice on HFD or NC diet. (G) Mean +SEM T_b_ during the active phase (dark phase) and the rest phase (light phase) of SPF and GF mice housed in LD and provided with HFD or NC diet. *P<0.05, **P<0.01, ***P<0.001 vs SPF mice. #P<0.05 v GF mice. LMA: SPF-NC n=18, SPF-HFD n=8, GF-NC n=16, GF-HFD n=7-8. T_b_: SPF-NC n=18, SPF-HFD n=8, GF-NC n=15, GF-HFD n=7. *P<0.05, **P<0.01, ***P<0.001 v SPF-NC; #P<0.05, ##P<0.01, ###P<0.001 v GF-NC.

**Table S1.**


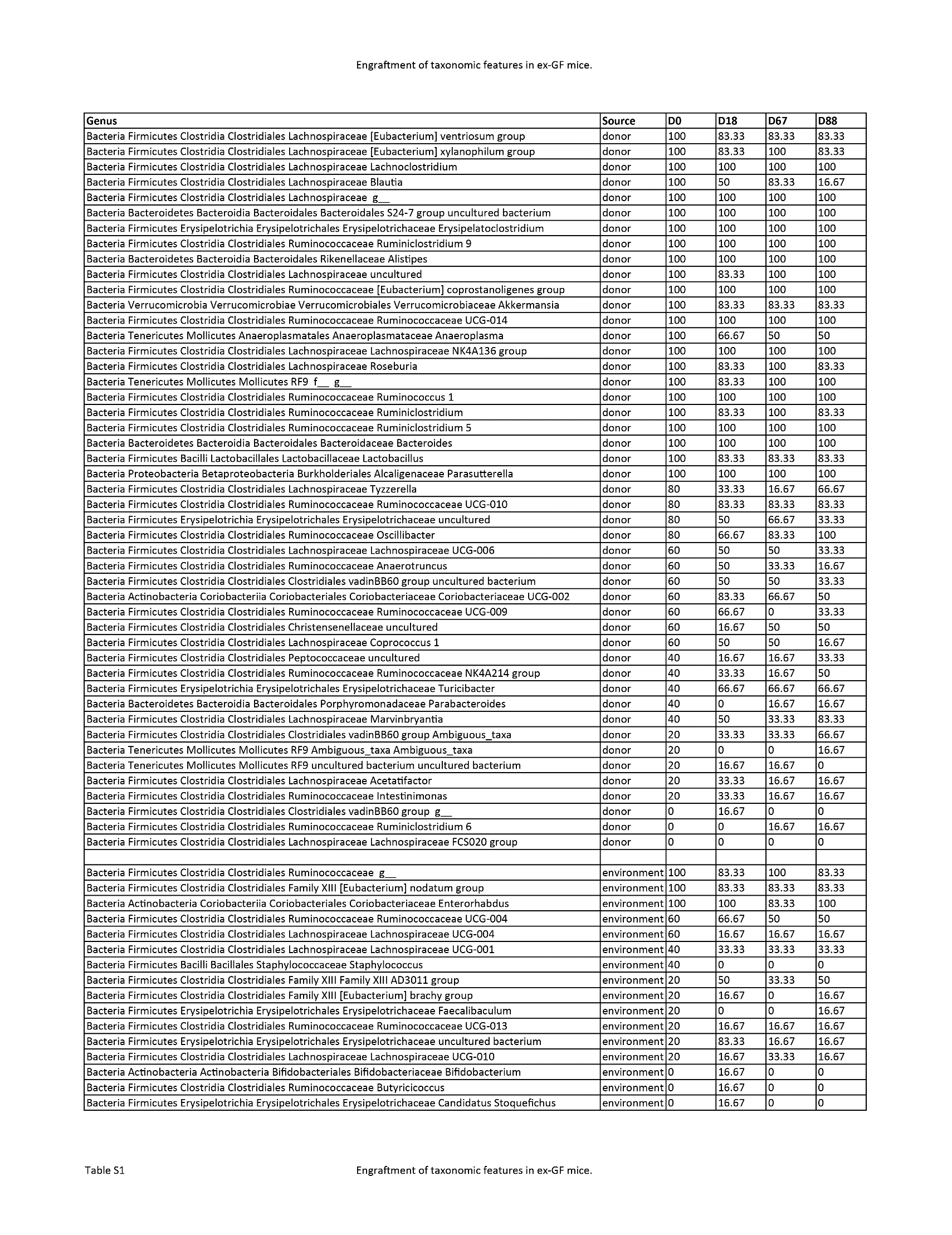


**Table S1.** **Engraftment of taxonomic features in ex-GF mice.** Columns list the percentage of ex-GF mice in which each taxonomic feature appeared across time points. Taxonomic features were grouped at the genus level. Features not present in donor sample were presumed to be derived from the environment since ex-GF mice were conventionally housed outside of the flexible film isolators. *day* 0 (D0): *n*=5; *day* 18 (D18)*, day* 67 (D67), and *day* 88 (D88): *n*=6
